# Microbial evaluation of zirconia and titanium implants in the anterior mandibula: a randomized controlled clinical trial

**DOI:** 10.1038/s41598-026-54915-0

**Published:** 2026-06-03

**Authors:** Kristian Kniha, Konstantin J. Scholz, Eva Kohnert, Sibylle Bartsch, Marius Heitzer, Frank Hölzle, Ali Al-Ahmad, Ali Modabber

**Affiliations:** 1 Department of Oral and Cranio-Maxillofacial Surgery, University Hospital RWTH Aachen, Pauwelsstraße 30, Aachen, Germany; 2 Private Dental Clinic Dres, Rosental 6, 80331 Kniha, Munich, Germany; 3 Institute for Medical Biometry and Statistics, Faculty of Medicine, University of Freiburg, Freiburg im Breisgau, Germany; 4 Center for Dental Medicine, Department of Operative Dentistry and Periodontology, Faculty of Medicine and Medical Center, University of Freiburg, Freiburg, Germany; 5 Department of Oral and Cranio-Maxillofacial Surgery, Aachen University, Pauwelsstraße 30, Aachen, 52074 Germany

**Keywords:** Dental implant, Ceramic, Zirconia, Titanium, Removable denture, Microbiota, Diseases, Health care, Medical research, Microbiology

## Abstract

To evaluate the effect of the implant material—either titanium or zirconia—on the development of bacterial deposits. In this one-year prospective split-mouth study, 20 patients with an edentulous lower jaw were treated with two zirconia and two titanium implants. Clip attachments were used for the removable denture. During the follow-up period, fluid samples were taken from around each patient’s implants and natural teeth to analyze the microbiota using DNA isolation, amplicon sequencing, and downstream analysis. Between the different time points, for the titanium material, the relative abundance of Actinomyces israelii increased significantly (p = 0.009) after 12 months when compared with the baseline. When comparing the different time points for each material, a significant decrease in the phyla Fusobacteria (p = 0.03) and Proteobacteria (p = 0.03) after six months versus baseline was detected for only zirconia, whereas the abundance of Firmicutes was significantly increased (p = 0.03). When comparing the different materials at each time point, the phylum Actinobacteria was found to be less abundant after 12 months on the zirconia implants than on the titanium implants (p = 0.04). By contrast, on the zirconia material, the relative abundance of the genus Leptotrichia decreased significantly after six months when compared with the baseline, while no significant changes in terms of this genus were found for the titanium implants or teeth over time. Zirconia tended to show less abundant bacterial deposits over time. The microbial diversity was lower on the titanium implants than on the ceramic material after 12 months. In addition, after 12 months of evaluation, neither the teeth nor the implants showed similar prevalences or levels of the target species.

**Supplementary Information:**

The online version contains supplementary material available at 10.1038/s41598-026-54915-0.

## Introduction

Dental implants have revolutionized modern dentistry by providing a durable, functional, and aesthetically pleasing solution for replacing missing or damaged teeth^1^. The materials used to produce dental implants play a crucial role in their success, with titanium and ceramic being two of the most widely used and well-researched materials applied in implantology.

Titanium has been available since the 1960 s and are now established as the gold standard, supported by the literature showing 10-year survival rates of over 90% thanks to its remarkable properties^2^. As a biocompatible material, titanium integrates seamlessly with bone through a process known as osseointegration, which ensures that the implant remains securely anchored^3^. Titanium is also known for its high strength, resistance to corrosion, and ability to withstand the mechanical forces of chewing and biting^4^, while its excellent biocompatibility reduces the risk of rejection or inflammation^5^. However, the metallic appearance of titanium implants, while hidden beneath the gumline, may not always meet aesthetic demands, especially in patients with thin gums or those who require implants in visible areas^6^.

By contrast, ceramic implants represent an alternative material with growing popularity, particularly among patients who are seeking a more natural-looking solution^7^. Made from zirconia, ceramic implants are known for their tooth-like appearance, which renders them an attractive option for those concerned about aesthetics. Ceramic implants are also biocompatible and resistant to corrosion, although they are generally less fracture-resistant than titanium and so may not be as suitable for high-stress areas such as the molar region^8^. Moreover zirconia implants have the advantage of less content release to the surrounding tissues^9^.

Both titanium and ceramic implants have distinct properties that impact their performance. For example, bacterial adhesion to implant surfaces is a critical factor influencing the long-term success of dental implants^10^. Indeed, implants can become a breeding ground for bacteria, which can lead to peri-implantitis (inflammation around the implant) and even implant failure^11^. Recent research in the field of dental implantology has focused on improving both the materials and the surface properties of the implants to enhance their performance and longevity^12^. Zirconia, as the ceramic material most commonly used in dental implants, has been the subject of extensive research aimed at enhancing its mechanical properties without compromising its aesthetic advantages^13^; however, because of the rather new material studies regarding zirconia implants and removable dentures remain scarce. To address this gap in the literature, this study primarily sought to evaluate the effect of the implant material—either titanium or zirconia—on the development of bacterial deposits in patients with a removable denture in the lower jaw. The null hypothesis of the present study was that there are no differences in microbial composition and diversity between titanium and zirconia implants over time. This study primarily provides a comparative analysis of the peri-implant microbiome associated with zirconia and titanium implants. While differences in microbial composition were observed, these findings should not be interpreted as demonstrating clinical superiority of zirconia or titanium implants.

## Materials and methods

In this one-year prospective split-mouth study, 20 volunteers who were entirely edentulous in the lower jaw were recruited. The exclusion criteria for this study were systemic disease (e.g., uncontrolled diabetes), smoking, untreated periodontitis, gingivitis, and severe bruxism or clenching habits.

**Fig. 1:**
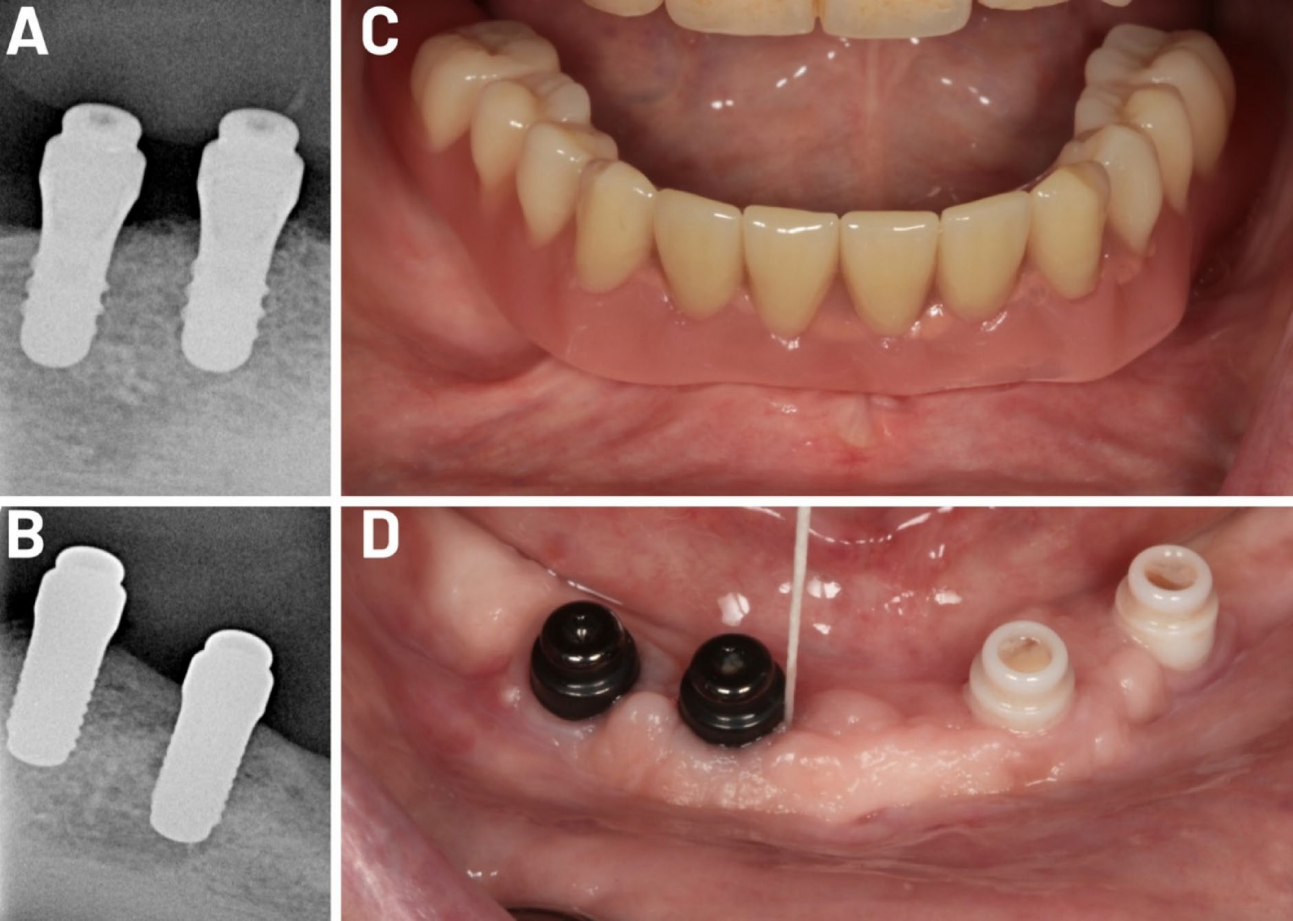
Radiologic presentation of a case after one year of follow-up. (**A**) Radiologic control images of titanium implants. (**B**) Radiologic control images of ceramic implants. (**C**) The removable prosthesis was checked for complications and function. (**D**) Clinical view of the implants.

Using the standard two-stage surgical procedure (e.g., first, unloaded implant healing, and then after three months, implant loading), four implants—two zirconia (PURE Ceramic Implant, 4.1 mm diameter implant, Straumann GmbH, Freiburg, Germany) and two titanium (TL SLactive, 4.1 mm diameter implant, Institut Straumann AG, Basel, Switzerland)—were placed in the mandibular inter-foramina region in each patient. Clip attachments (PUREloc and Novaloc locators, Institut Straumann AG, Basel, Switzerland) were used to secure each implant (Fig. 1). The balanced computerized randomization approach to determining the individual implant position was used to select the side where the titanium or ceramic implants were inserted (either the right or left side for two zirconia or ceramic implants). The implants, which were inserted by one surgeon according to the relevant manufacturer’s protocol, had a diameter of 4.1 mm, a minimum length of 8 mm, and a maximum length of 12 mm.

After insertion, there was a 12-month follow-up period. The evaluation focused on bacterial samples, which were analyzed after 3 months (T0, two-stage after 3 months osseointegration), 6 months (T1), and 12 months (T2) following insertion to characterize the bacterial communities and microbiota diversity. Samples were also taken from natural teeth in the upper jaw to compare the outcomes between implants and teeth. During the study period, the patients were instructed to perform optimal oral hygiene procedures (e.g., brushing, flossing, rinsing). Before sampling, patients were instructed not to rinse their mouth, or use antimicrobial mouthwash on the day of sampling. They were also asked to avoid eating or drinking shortly before sample collection to minimize immediate dietary contamination. No prolonged fasting period was required. Patients who had taken antibiotics within the preceding period were excluded from sampling.

We confirm that the experimental protocol was approved by Ethics Committee at the Faculty of Medicine of RWTH Aachen University (RWTH Aachen) EK 180/19.

The primary outcome of this study was the longitudinal change in peri-implant microbial composition and diversity associated with titanium and zirconia implants. Microbial composition was defined as the relative abundance of bacterial taxa at species, genus, and phylum levels, as determined by 16 S rRNA gene sequencing. Microbial diversity was assessed using alpha diversity indices, including the Shannon–Weiner index, richness, and Pielou’s evenness.

The primary analysis metrics included (i) changes from baseline to 6 and 12 months and (ii) differences between implant materials at each time point. Data were aggregated as mean values and distributions (boxplots) for diversity indices and relative abundances.

Secondary outcomes included the identification of differentially abundant taxa over time and between materials, as well as beta diversity differences assessed using Bray–Curtis dissimilarity and non-metric multidimensional scaling. These outcomes were analyzed using mixed-effects models and multivariate statistical approaches.

All outcomes were assessed at predefined time points: 3 months (T0), 6 months (T1), and 12 months (T2) after implant placement.

### DNA isolation and amplicon sequencing

DNA was isolated from the samples using the DNeasy Blood and Tissue Kit (Qiagen, Hilden, Germany), as previously described^14^. A mock community was used as the positive control, as described by Anderson et al.^15^, which included the following microbial species: *Fusobacterium nucleatum* (ATCC 25586), *Streptococcus mutans* (DSM 6178), *Streptococcus sanguinis* (DSM 20068), *Streptococcus mitis* (ATCC11843), *Veillonella parvula* (DSM 2008), *Parvimonas micra* (ATCC 33270), *Actinomyces odontolyticus* (DSM 19120), *Neisseria flavescens* (DSM 17633), *Tannerella forsythia* (ATCC 43037), and *Porphyromonas gingivalis* (W381). The DNA amplicon sequencing was performed by Eurofins Genomics (Konstanz, Germany) using the V1–V3 region of the 16 S rRNA gene sequence.

### Quality control, removal of host contamination, and taxonomy assignment

In terms of bioinformatics, paired-end 16 S rRNA gene sequencing data were processed using the QIIME2 framework (Caporaso Lab, Northern Arizona, USA). The raw sequence data, provided in FASTQ format, were first imported into QIIME2 as a.qza artifact using the qiime tool’s import function. The sequence quality was assessed with the qiime demux summarize function. The DADA2 algorithm (qiime dada2 denoise-paired) was employed for quality filtering, chimera removal, and paired-end sequence denoising, with the forward reads truncated at 320 bp and the reverse reads truncated at 242 bp due to a rapid drop off in the quality score. A feature table was generated, and summary statistics were computed using the qiime feature-table summarize and qiime feature-table tabulate-seqs commands.

For the phylogenetic analysis, the sequences were aligned using MAFFT, and a phylogenetic tree was constructed using FastTree via the qiime phylogeny align-to-tree-mafft-fasttree pipeline. Taxonomic classification was performed using the SILVA 138 reference database. More specifically, a custom classifier was trained using the qiime feature-classifier fit-classifier-naive-bayes function based on the SILVA 138 99% operational taxonomic unit reference sequences, with the amplicons extracted based on the forward (AGRRTTYGATYMTGGCTCAG) and reverse (TBACCGCGGCTGCTGGCAC) primers. Classification of the sequences was then performed with the trained classifier using the qiime feature-classifier classify-sklearn function.

### Downstream analysis

All the downstream analyses were conducted in RStudio (R version 3.6.). Data were imported into a phyloseq object from the phyloseq package in R. The results are presented here at the species level. Additionally, a genus level dataset was created by agglomerating the data at the genus level. For all the statistical tests, a significance level of 5% was applied.

To characterize the bacterial communities within a given sample, the α-diversity was analyzed. The Shannon–Weiner diversity index, richness, and Pielou’s evenness index were calculated as measures of the α-diversity using the vegan package. The results are presented as boxplots constructed using the ggplot2 package. To test for differences in the alpha diversity between time points and materials, a linear mixed effects model was employed, taking into account the paired nature of the data.

The microbial similarity between samples was investigated using the β-diversity. The phyloseq package was used to calculate the Bray–Curtis distances for the dissimilarity measurement and to plot the results using non-metric multidimensional scaling. A pairwise PERMANOVA with 999 permutations based on the adonis function in the vegan package was applied to test for differences in the β-diversity between time points.

To identify the most appropriate statistical test with which to detect differentially abundant taxa, the following evaluation pipeline was followed. First, 10 synthetic datasets were generated using the metaSPARSim simulation tool, which was calibrated based on experimental data to closely resemble the original conditions. More specifically, the proportion of zeros and the prevalence of differentially abundant taxa were adjusted to ensure that the synthetic data mirrored the characteristics of the original dataset. Next, 14 statistical tests were applied to the following synthetic datasets: ALDEx2, ANCOM-BC, corncob, DESeq2, edgeR, LEfSe, limma voom (TMM), limma voom (TMMwsp), MaAsLin2, MaAsLin2 (rare), metagenomeSeq, t-test (rare), Wilcoxon test (CLR), and Wilcoxon test (rare). To evaluate the performance of each test, the sensitivity and specificity were calculated, and receiver operating characteristic (ROC) curves were plotted. The statistical test with the highest sensitivity at a significance threshold of 0.05 was selected, with MaAsLin2 emerging as the best-performing test.

Within the MaAsLin2 framework, a mixed effects model was employed to account for the paired nature of the data. This model was used to identify species, genera, and phyla that were differentially abundant across time points and between materials. A 10% prevalence threshold was applied, and the data were AST-transformed. To control for multiple comparisons, the Benjamini–Hochberg correction was applied. For all the taxa that showed significant changes, boxplots were generated using the ggplot2 package for visualization purposes.

To assess the robustness of the findings, a sensitivity analysis using bootstrapping with 200 repetitions was performed. For each taxon, the mean effect size and the corresponding 95% confidence interval (CI) were calculated. Taxa with bootstrap confidence intervals that did not cross zero were considered to have satisfied the sensitivity analysis, indicating a robust result.

Peri-implant clinical parameters, including probing depth, bleeding on probing (BOP), and plaque index, were recorded throughout the study period; however, these data are part of a separate analysis and are not included in the present report, which focuses on microbiological outcomes.

### Statistical analysis

The sample size was calculated using G*Power software (G*Power, version 3.1.9.2, Düsseldorf, Germany, Faul et al.^16,17^). The matched pairs t-test was used as an indication. It was hypothesized that a lower bacterial load leads to less soft tissue inflammation (at 12 months after insertion), as assessed by bacterial sampling. Using a 0.05 significant level, a mean of differences of 0.35/standard deviation of 0.5 after^18^, an effect size of 0.71, and a power of 80% after Cohen, a total of 18 participants were required to verify the (patient-based) hypothesis. Based on an expected drop-out rate of 8%, the sample size was increased from 18 to 20.

## Results

### Microbiota diversity

A total of 20 patients received dental implants in this study. Among these patients, only 18 attended the one-year follow-up assessment due to two patients’ refusal to appear at the clinic. In this study, the supragingival plaque was analyzed in comparison to the dental plaque on natural teeth at three different time points—namely, 3 months (T0), 6 months (T1), and 12 months (T2) after implantation. The diversities and (dis) similarities of the patients’ microbiomes were characterized.

**Fig. 2:**
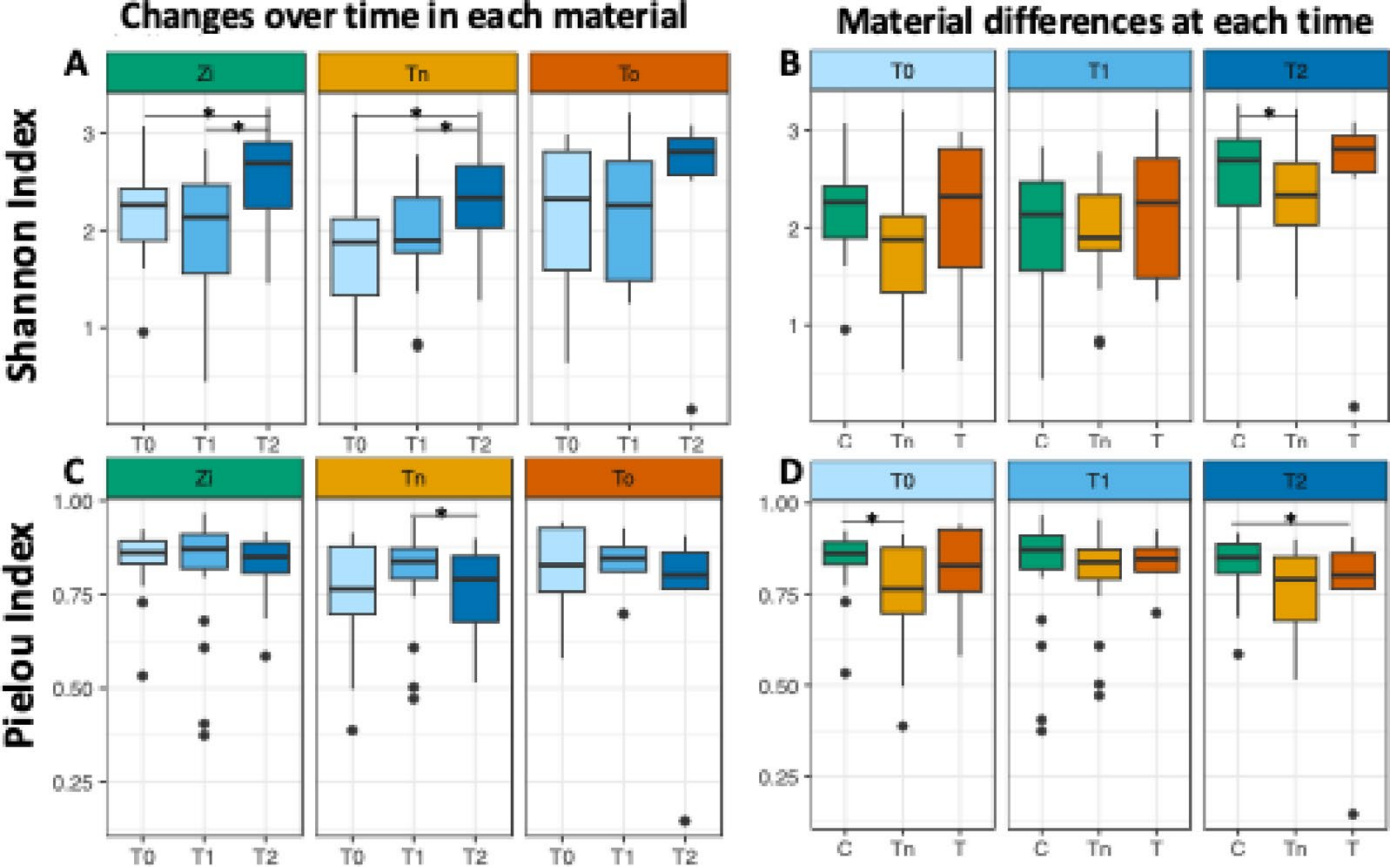
The α-diversity quantified by the Shannon index and Pielou index. While the Shannon index takes the number of species and their abundance into account, the Pielou index quantifies how evenly the species are distributed. Asterisks indicate significant differences based on a p-value < 0.05, as obtained by a linear mixed effects model. (**A**) For the zirconia (Zi), titanium (Tn), and tooth (To) samples, the Shannon index at baseline (T0), 6 months after implantation (T1), and 12 months after implantation (T2) is displayed, showing an increase in α-diversity over the course of the study. In the zirconia and titanium samples, this increase is significant. (**B**) The boxplot shows the same data as in (**A**), only rearranged to compare the materials at the three different time points. At T2, the titanium samples show decreased diversity when compared with zirconia. (**C**) The evenness of the bacterial species remains relatively constant over time in all the materials. Only in the titanium samples can a decrease from T1 to T2 be observed. (**D**) Comparison of the species evenness between the different materials. At baseline, the titanium samples have less evenly distributed species than zirconia. At the end of the study, the tooth samples have less evenly distributed species than zirconia.

The α-diversity was examined using two indices: the Shannon index, which describes the species richness, and the Pielou index, which describes the evenness of the various biofilm samples. On both the ceramic and titanium implants, the microbial diversity increased significantly after 12 months when compared with the baseline and T1, while the microbial diversity on the natural teeth did not change significantly over the entire study period (Fig. 2A). When considering the materials at each time point, the microbial diversity was significantly lower on the titanium implants than on the ceramic material at T2 (Fig. 2B). The evenness of the microbial communities was only significantly lower on the titanium implants after 12 months than after 6 months (Fig. 2C). At T0, the evenness on the titanium implants was significantly lower than on the ceramic implants, while at T2, the evenness on the natural teeth was significantly lower than on ceramic implants.

**Fig. 3:**
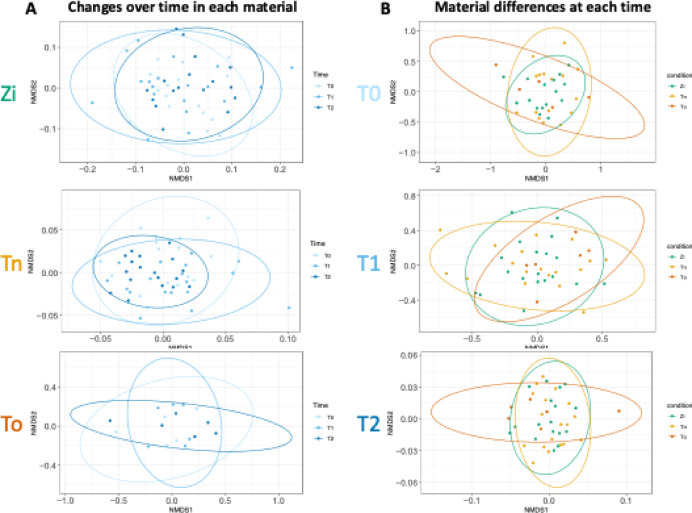
The β-diversity: Non-metric multidimensional scaling plots based on the Bray–Curtis index. Each point represents a single sample. (**A**) For the zirconia (Zi), titanium (Tn), and tooth (To) samples, there is no systematic change in β-diversity between baseline (T0), 6 months after implantation (T1), and 12 months after implantation (T2). (**B**) The boxplot shows the same data as in (**A**), only rearranged to compare the materials at the three different time points. During the course of the study, no systematic change between the materials can be observed. To test for differences in the β-diversity between materials or time points, a pairwise PERMANOVA test was applied, yielding non-significant results.

To characterize the differences and similarities in the microbiota between the materials and time points, we calculated the β-diversity based on the Bray–Curtis distance. In Fig. 3, non-metric multidimensional scaling plots are shown to identify the clustering patterns of the samples in order to reveal the dissimilarities in each material across all time points (Fig. 3A) and among the materials at each time point (Fig. 3B). The results reveal similar clustering within the samples of each material at all time points, indicating the microbial community of each material to have no dependence on the time point (Fig. 3A). No significant differences in the microbial communities between the materials at each time point were observed (Fig. 3B).

### Microbial community composition

**Fig. 4:**
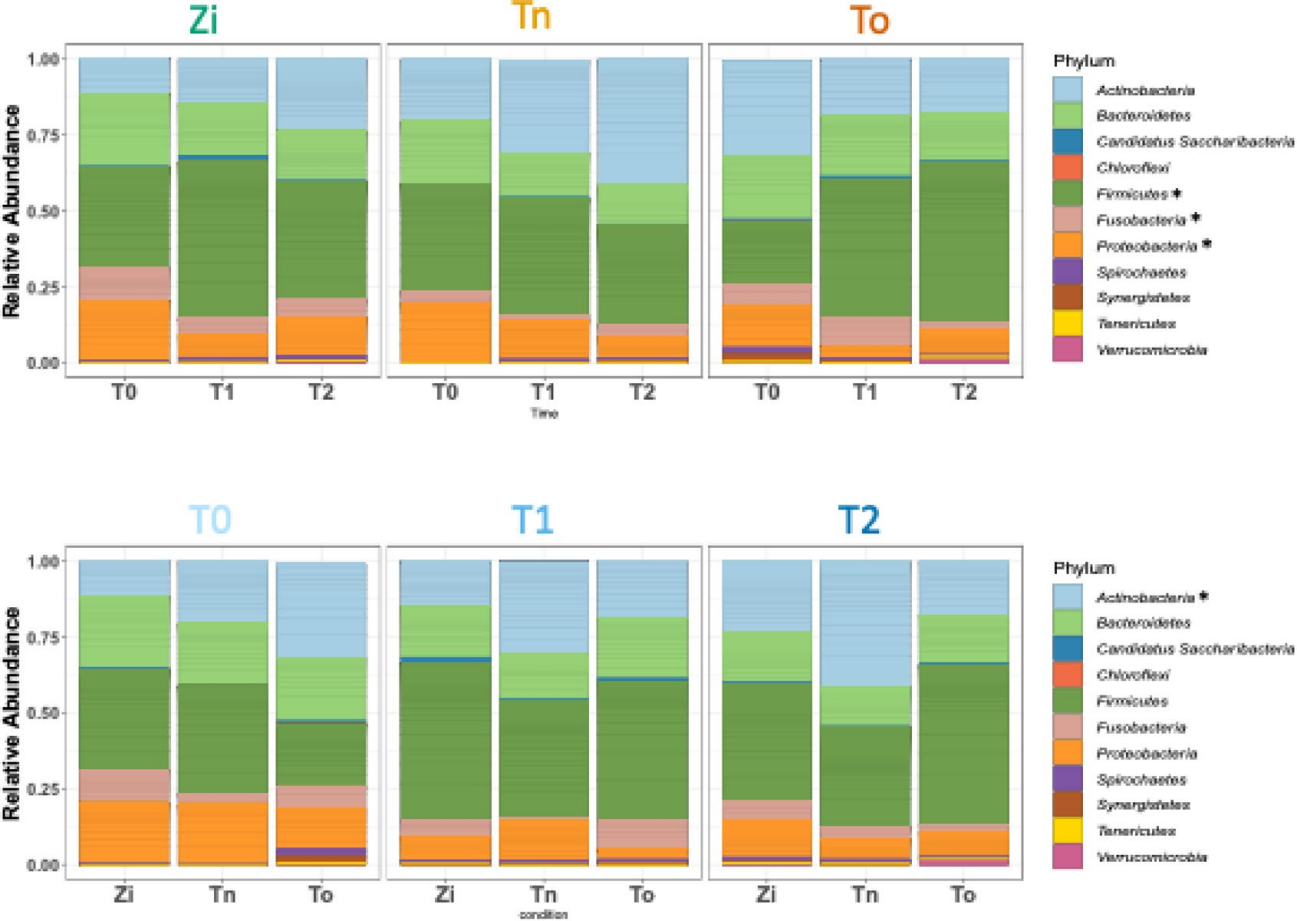
Relative abundance at the phylum level. (**A**) Change in the phyla composition over time for the different implant materials and for tooth (To). For the zirconia (Zi) samples, Fusobacteria and Proteobacteria significantly decreased from T0 to T1 (*p* = 0.031, *p* = 0.035), while Firmicutes increased from T0 to T1 (*p* = 0.032). (**B**) Comparison of the phyla composition between the different materials at each time point of the study. For T2, Actinobacteria were less abundant in zirconia than in titanium (*p* = 0.04).

Overall, 138 different genera (Supplementary Table S1) and 399 species (Supplementary Table S2) were identified. The relative abundance of each species within the mock community, which was used as a positive control for the DNA extraction, was similar in the samples from each of the three materials (titanium, zirconia and Tooth, Supplementary Figure S1). Moreover, in the mock community, which was also used as a positive control for the DNA isolation and shotgun sequencing procedure, all the taxa could be detected, and taxa belonging to 11 phyla (Fig. 4) were detected on all the implant materials and at all time points. The main phyla identified in this study were Firmicutes (39.02 ± 25.29%), Actinobacteria (24.03 ± 22.58%), Bacteroides (17.71 ± 16.2%), Proteobacteria (11.87 ± 15.05%), and Fusobacteria (5.06 ± 7.26%). The other detected phyla were Candidatus Saccharibacteria, Synergistetes, Chloroflexi, Spirochaetes, Tenericutes, and Verrucomicrobia. In terms of the different time points for each material, a significant decrease in the phyla Fusobacteria (*p* = 0.03) and Proteobacteria (*p* = 0.03) after six months (T1) when compared with the baseline (T0) was detected only for zirconia, while the abundance of Firmicutes was significantly increased (*p* = 0.03). When comparing the different materials at each time point, the phylum Actinobacteria was less abundant after 12 months (T2) on the zirconia implants when compared with the titanium implants (*p* = 0.04).

**Fig. 5:**
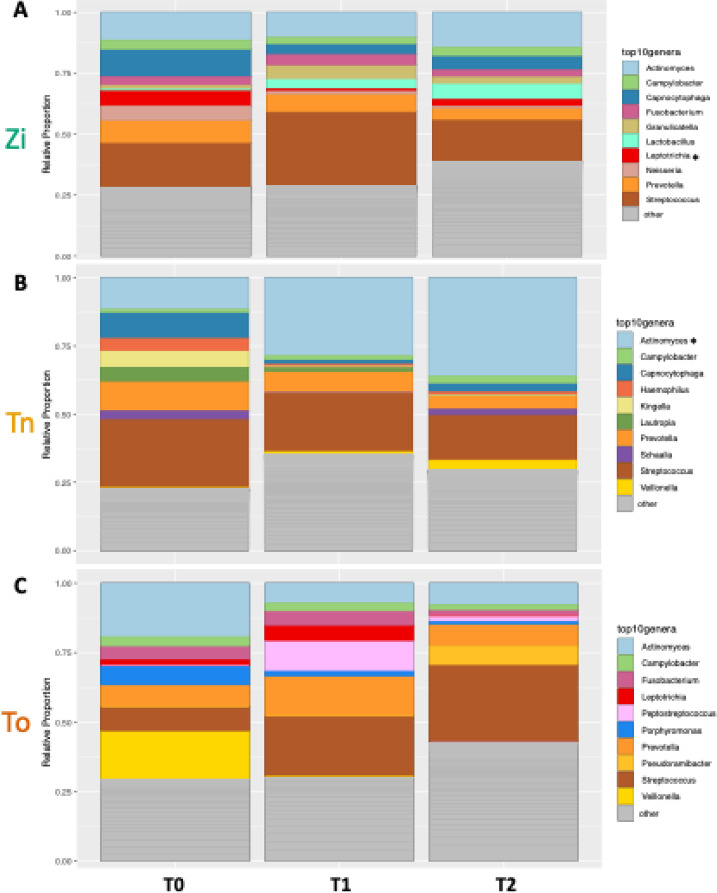
Barplots showing the relative abundance of the 10 genera with the highest mean abundance across all the samples. The remaining genera are summarized in the “other” category. A linear mixed effects model was applied to test for differences between time points. (**A**) For the zirconia (Zi) samples, *Leptotrichia* showed a significant decrease at T1 when compared with T0 (*p* = 0.02). (**B**) In the titanium (Tn) samples, no significant genera changes were detected over time. (**C**) In the tooth (To) samples, no significant genera changes were detected over time.

When looking at the genera with the highest relative abundance, it can be seen that the top 10 genera are different for the different implant surfaces (Fig. 5). The following top 10 genera were detected on the zirconia (Zi) implants: *Actinomyces* (12.32 ± 16.29%), *Campylobacter* (3.40 ± 5.11%), *Capnocytophaga* (7.33 ± 11.72%), *Fusobacterium* (3.62 ± 5.90%), *Granulicatella* (3.70 ± 10.45%), *Lactobacillus* (3.57 ± 11.20%), *Leptotrichia* (3.50 ± 6.45%), *Neisseria* (2.50 ± 6.58%), *Prevotella* (7.33 ± 9.24%), and *Streptococcus* (22.99 ± 24.08%). When compared with T0, the abundance of *Leptotrichia* on the zirconia implants decreased significantly after six months (*p* = 0.01).

The following top 10 genera were found on the titanium (Tn) implants: *Actinomyces* (25.63 ± 27.39%), *Campylobacter* (1.74 ± 4.30%), *Capnocytophaga* (4.94 ± 10.10%), *Haemophilus* (2.17 ± 6.53%), *Kingella* (2.13 ± 9.52%), *Lautropia* (2.33 ± 11.68%), *Schaalia* (1.64 ± 4.78%), *Veillonella* (1.88 ± 3.40%), *Prevotella* (8.11 ± 14.97%), and *Streptococcus* (21.78 ± 1.62%). No significant changes were detected over time with regard to the genera on the titanium implants.

On the natural teeth (To), the following top 10 genera were detected: *Actinomyces* (11.48 ± 16.68), *Campylobacter* (2.47 ± 4.07%), *Fusobacterium* (4.52 ± 5.29%), *Leptotrichia* (2.37 ± 6.24%), *Peptostreptococcus* (4.06 ± 10.20%), *Porphyromonas* (3.12 ± 9.70%), *Prevotella* (9.63 ± 13.01%), *Pseudoramibacter* (2.37 ± 6.90%), *Streptococcus* (22.00 ± 31.115%), and *Veillonella* (6.14 ± 22.42%). No significant changes were detected over time in terms of the genera on the natural teeth.

**Fig. 6:**
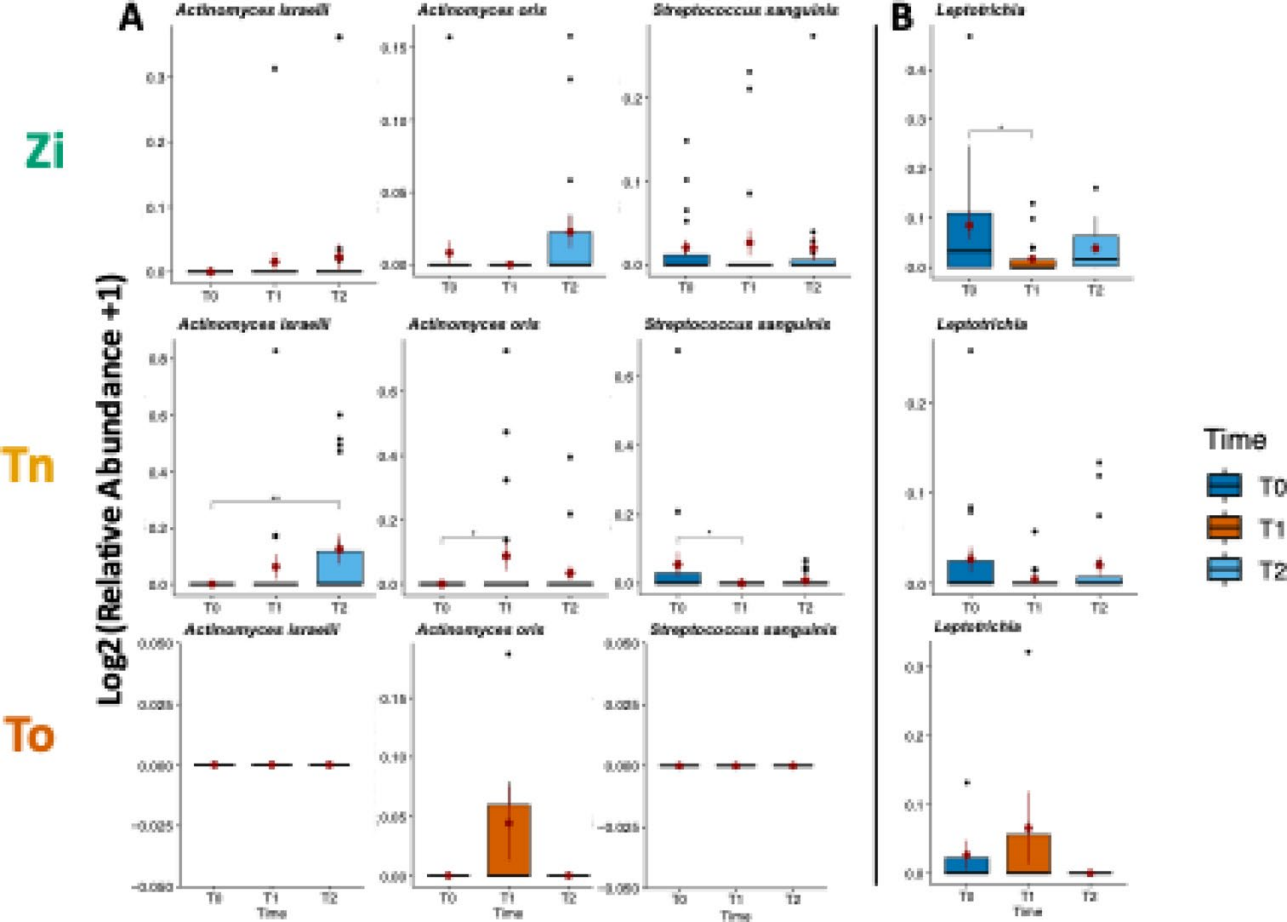
Boxplots showing the distribution of all the species and genera that were identified as changing significantly between time points or materials. The relative abundance values were log2-transformed after adding 1 to account for 0 values. Red dots indicate the mean value, while red lines indicate the corresponding standard error of the mean. (**A**) Significantly changed species. All the significant changes were observed within the titanium (Tn) samples. *Actinomyces israelii* showed a significant increase in abundance at the end of the study when compared with baseline (p-adjusted = 0.009). *Actinomyces oris* showed an increase after six months when compared with baseline (p-adjusted = 0.039), while *Streptococcus sanguinis* decreased in relative abundance (p-adjusted = 0.033). No significant changes were detected for the tooth (To) samples. (**B**) Significantly changed genus. *Leptotrichia* decreased after six months when compared with baseline. The decrease was significant for the zirconia (Zi) samples (p-adjusted = 0.022). No significant changes were detected for the tooth (To) samples.

Figure 6 shows the bacterial species for which significant differences were found between the different time points for the titanium (Tn) implant material. These species included *Actinomyces israelii*, whose relative abundance increased significantly (*P* = 0.009) after 12 months (T2) when compared with the baseline (T0). The relative abundance of *Actinomyces oris* increased significantly (*p* = 0.039) at T1 when compared with T0, while the relative abundance of *Streptococcus sanguinis* decreased significantly (*p* = 0.033) at T1 when compared with T0. On the zirconia (Zi) material and on the natural teeth (To), the abundance of the aforementioned three species did not change significantly (Fig. 6). On the contrary, on the zirconia material, the relative abundance of the genus *Leptotrichia* decreased significantly after six months (T1) when compared withT0, while no significant changes in this genus were found for the titanium implants or the natural teeth over time.

## Discussion

This study mainly presents a comparative assessment of the peri-implant microbiome in relation to zirconia and titanium implants. Although variations in microbial composition were identified, the results should not be understood as evidence that either zirconia or titanium implants are clinically superior. This study compared the composition and diversity of the oral microbiota associated with zirconia implants, titanium implants, and natural teeth. It found significant differences in the microbial profiles among the three surfaces, highlighting the complex interactions between the host tissue, biomaterial properties, and microbial environment.

Microorganisms play a central role in peri-implant health, as the composition and maturation of the biofilm are key determinants in the transition from health to disease. Early colonizers such as *Actinomyces* spp. contribute to initial biofilm formation, creating a foundation for more complex microbial communities^19^. In contrast, taxa such as *Fusobacteria*and certain *Proteobacteria* are often associated with biofilm maturation and have been linked to inflammatory processes and peri-implant disease progression. Therefore, even subtle shifts in microbial composition may influence the ecological balance at the implant surface and potentially affect long-term tissue stability.

In this context, the present study is important because it provides longitudinal, in vivo data on how different implant materials may influence microbial colonization patterns^20^. By using a split-mouth design, inter-individual variability was minimized, allowing for a more direct comparison between titanium and zirconia surfaces. Although the clinical implications cannot be definitively established, the observed material-dependent differences in microbial profiles contribute to a better understanding of how implant surface characteristics may modulate the peri-implant microbiome. This may ultimately support the development of biomaterials with improved biological performance and reduced susceptibility to biofilm-associated complications.

The microbial communities around natural teeth exhibited the highest species richness and diversity, which is consistent with the presence of a complex and mature biofilm developed over time within the natural periodontium. By contrast, both the zirconia and titanium implants harbored less diverse microbiota, which aligns with prior reports suggesting that peri-implant niches host a less complex but potentially more pathogenic biofilm^21^. This finding is in line with the results of another study regarding the association between peri-implant/periodontal health and peri-implantitis/periodontitis^22^. Moreover, the incidence of microorganisms in the teeth was found to be greater than in the implants.

Notably, the zirconia implants demonstrated a distinct microbial profile when compared with the titanium implants. The relative abundance of pathogenic anaerobes such as *Porphyromonas gingivalis*, *Treponema denticola*, and *Tannerella forsythia*—collectively known as the “red complex”—was lower in the zirconia sites. This finding supports the hypothesis that zirconia surfaces may be less conducive to the adhesion and maturation of periodontal pathogens, possibly due to the lower surface free energy and smoother surface topography^23,24^. This finding accords with the results of another study, which found that the adhesion of bacteria to ZrO2 implants was reduced when compared with Ti implants and concluded that ZrO2 implants might contribute to reduced biological complications such as peri-implantitis^25^. Titanium implants, while highly valued for their osseointegration properties, are associated with a higher prevalence of pro-inflammatory bacterial species. This might contribute to explaining the higher incidence of peri-implantitis observed in some titanium-based systems^25^. Titanium’s susceptibility to corrosion and potential release of metal ions may also contribute to the local inflammatory response and biofilm dysbiosis^26^.

In the present study, at the 12-month time point (T2), the phylum *Actinobacteria* was less abundant on the zirconia implants than on the titanium implants (*p* = 0.04). By contrast, the zirconia implants showed a significant reduction in the relative abundance of the genus *Leptotrichia* after six months (T1) when compared with baseline (T0), whereas no significant temporal changes in this genus were observed for the titanium implants or natural teeth.

The lower bacterial load and inflammatory potential observed around the zirconia implants suggest a possible clinical advantage in terms of maintaining peri-implant tissue health. However, the long-term implications of these differences in the microbiota composition remain to be fully understood, especially in relation to implant longevity, patient immune response, and prosthetic function^27^. Importantly, while zirconia might favor a less pathogenic microbiota, the observed functional differences may be influenced by other variables, such as the implant design, surface modification, oral hygiene habits, and patient-specific factors^28^. Additionally, the similarity in the microbiota between implants and natural teeth in patients with a healthy periodontium underscores the role of both systemic and behavioral factors in microbial colonization^29^.

Based on the observed differences in microbial composition and diversity between titanium and zirconia implants over time, the null hypothesis was rejected. Natural teeth were included as a control group, as they represent the clinical reference for fixed dental rehabilitation, with implant therapy aiming to mimic the function and biological behavior of the natural dentition. Therefore, their inclusion provides a meaningful benchmark for comparison. However, it must be acknowledged that natural teeth and implants differ fundamentally in their anatomical and biological characteristics, including the presence of the periodontal ligament and differences in soft tissue attachment. These factors may influence the local microbial environment, and thus direct comparisons should be interpreted with caution. In addition to material composition, other factors may have influenced biofilm development in the present study. Salivary flow plays a crucial role in microbial clearance and nutrient supply, and variations in salivary distribution between mandibular implant sites and other intraoral locations may have affected microbial colonization patterns. Furthermore, the use of overdentures with clip attachments may create specific niches that promote plaque accumulation and alter biofilm maturation due to limited self-cleansing and mechanical access. Surface characteristics of implant materials, particularly surface roughness (Ra), are also known to influence bacterial adhesion. Although both titanium and zirconia implants are manufactured to achieve clinically acceptable surface properties, even minor differences in roughness may affect initial microbial attachment and subsequent biofilm development.

Finally, limitations inherent to 16 S rRNA gene sequencing should be considered. While this method allows for comprehensive profiling of microbial communities, it has limited resolution at the species level and does not provide direct information on microbial function or virulence. The present study identified differences in peri-implant microbial composition associated with zirconia and titanium implants. However, these findings should be interpreted cautiously, as potential influencing factors such as salivary flow, overdenture design, implant surface characteristics, and the limitations of 16 S rRNA sequencing were not directly evaluated as causal determinants. Therefore, the findings of the present study should be interpreted as descriptive of compositional changes rather than definitive indicators of functional or pathogenic differences.

All implants were restored with overdentures, which may influence biofilm accumulation due to specific retention elements and potentially reduced self-cleansing. Patient-dependent oral hygiene practices may have further affected microbial colonization patterns. Furthermore, the present analysis primarily reflects microbial composition without distinguishing between supragingival and subgingival biofilm compartments, which may differ in their relevance for peri-implant health and disease progression. As peri-implantitis is predominantly associated with subgingival dysbiosis, this should be taken into account when interpreting the findings. From a mechanistic perspective, differences between zirconia and titanium may be partly explained by material-related surface properties, such as surface energy and wettability, which can influence initial bacterial adhesion and biofilm formation. Zirconia surfaces have been suggested to exhibit lower bacterial affinity in certain contexts, potentially contributing to the reduced bacterial colonization observed in the present study.

A further potential limitation of the present study is the use of a split-mouth design, which may allow for intraoral cross-contamination between implant sites. Although this approach reduces inter-individual variability and strengthens the comparability between materials, the oral cavity represents a shared microbial environment, and bacterial transmission between sites cannot be fully prevented. The shared oral environment may facilitate microbial exchange between sites, and the overdenture design may influence plaque accumulation and microbial composition around both implant types. In addition, the use of natural teeth as a control introduces biological differences between teeth and implants that may affect direct comparisons. Therefore, the observed microbiome differences should be interpreted carfully, as some degree of microbial overlap may have occurred. Future studies employing next-generation sequencing and longitudinal monitoring will be essential to clarify the temporal dynamics of biofilm formation on implants made of different materials. Furthermore, personalized risk assessment models incorporating host–microbiome interactions could improve implant success rates and guide implant material selection in clinical practice. Although this study identified differences in microbial abundance and diversity between zirconia and titanium implant sites, these findings should be interpreted with caution in terms of clinical relevance. The present study was not designed to determine whether these microbial shifts translate into direct clinical benefits, influence implant material selection, or reduce the risk of peri-implantitis. Therefore, the observed microbiome differences should be regarded as exploratory and associative rather than evidence of clinical advantage. Future longitudinal studies combining microbiome analysis with clinical outcome parameters are needed to determine whether such microbial differences have implications for implant prognosis or peri-implant disease prevention.

## Conclusion

After 12 months of evaluation, neither the teeth nor the implant materials showed similar prevalences or levels of the target species. Zirconia tended to show less abundant bacterial colonization parameters over time. The microbial diversity was lower on the titanium implants than on the ceramic material after 12 months. The results revealed similar clustering within the samples of each material at all the time points. Within the limitations of this study, zirconia and titanium implants demonstrated distinct but not definitively superior microbial profiles; zirconia showed a tendency toward reduced bacterial colonization, while titanium exhibited lower microbial diversity, without clear evidence that one material is clinically superior to the other.

## Supplementary Information

Below is the link to the electronic supplementary material.

Supplementary Material 1

Supplementary Material 2

Supplementary Material 3

## Data Availability

All data generated for this study are available from the corresponding authors upon reasonable request.
