# Correction: Microbial evaluation of zirconia and titanium implants in the anterior mandibula: a randomized controlled clinical trial

**DOI:** 10.1038/s41598-026-60173-x

**Published:** 2026-06-30

**Authors:** Kristian Kniha, Konstantin J. Scholz, Eva Kohnert, Sibylle Bartsch, Marius Heitzer, Frank Hölzle, Ali Al-Ahmad, Ali Modabber

**Affiliations:** 1 Department of Oral and Cranio-Maxillofacial Surgery, University Hospital RWTH Aachen, Pauwelsstraße 30, Aachen, Germany; 2 Private Dental Clinic Dres, Rosental 6, 80331 Kniha, Munich, Germany; 3 Institute for Medical Biometry and Statistics, Faculty of Medicine, University of Freiburg, Freiburg im Breisgau, Germany; 4 Center for Dental Medicine, Department of Operative Dentistry and Periodontology, Faculty of Medicine and Medical Center, University of Freiburg, Freiburg, Germany; 5 Department of Oral and Cranio-Maxillofacial Surgery, Aachen University, Pauwelsstraße 30, Aachen, 52074 Germany

Correction to: *Scientific Reports* 10.1038/s41598-026-54915-0, published online 03 June 2026

The original version of this Article contained an error in the spelling of the author Konstantin J. Scholz which was incorrectly given as Konstantin Scholz.

The original Article has been corrected.

