# Supplementary material for: Microbial evaluation of zirconia and titanium implants in the anterior mandibula: a randomized controlled clinical trial
Source: Sci Rep. 2026 Jun 3;16:17031. doi: 10.1038/s41598-026-54915-0 (PMC13230837; doi:10.1038/s41598-026-54915-0)
Supplement: Supplementary file 1 — Supplementary Material 1 [file 41598_2026_54915_MOESM1_ESM.docx]

**Supplementary material**

**Sup. Table 1**

List of microbiological measurements for the tests performed

**Sup. Table 2**

Table of measurements for the tests analysed
