# Supplementary material for: Microbial evaluation of zirconia and titanium implants in the anterior mandibula: a randomized controlled clinical trial
Source: Sci Rep. 2026 Jun 3;16:17031. doi: 10.1038/s41598-026-54915-0 (PMC13230837; doi:10.1038/s41598-026-54915-0)
Supplement: Supplementary file 2 — Supplementary Material 2 [file 41598_2026_54915_MOESM2_ESM.pdf]

	Zirconium					
**Genus**	**T0_mean**	**T0_sd**	**T1_mean**	**T1_sd**	**T2_mean**	**T2_sd**
Abiotrophia	0.0064	0.02	0.0079	0.0356	0.0052	0.0199
Acetobacteroides	0	0	0	0	0	0
Acinetobacter	0	0	0	0	0	0
Actinomyces	0.1191	0.2039	0.1059	0.1354	0.1466	0.1482
Aerococcus	0	0	0	0	9e-04	0.0038
Aggregatibacter	0.0026	0.0067	0.0049	0.0165	0.0053	0.016
Alishewanella	0	0	0	0	0.0017	0.0072
Alkalibacterium	0.0124	0.0542	0.0034	0.0112	0.0063	0.0196
Alkaliflexus	0	0	0	0	0.0016	0.0066
Alkalihalobacillus	0	0	0	0	3e-04	0.0014
Alkalilacustris	0.0047	0.0156	0.001	0.0046	0	0
Alkalimonas	0	0	0	0	0.0049	0.0206
Alkaliphilus	0	0	0	0	8e-04	0.0025
Alloprevotella	0.0156	0.0493	0	0	0	0
Aminipila	0	0	0	0	0	0
Anaerobacillus	0	0	0	0	0.002	0.0069
Anaerobranca	0	0	0	0	4e-04	0.0018
Anaerocella	0	0	0	0	0	0
Anaerocolumna	0	0	0	0	0	0
Anaeroglobus	0.0017	0.0073	0	0	0.0016	0.0043
Anaeromassilibacillus	0	0	0	0	0	0
Anaerovorax	0	0	0	0	0	0
Atopobium	0.0028	0.0123	0.0031	0.014	0.0076	0.0323
Azospirillum	0	0	0	0	0	0
Bacillus	0.0022	0.0095	9e-04	0.0029	0.0143	0.0432
Bacteroides	0	0	0.0044	0.0196	0.0028	0.0117
Belliella	0	0	0	0	0	0
Bergeyella	0.0076	0.0192	0.0103	0.0317	0.0055	0.0099
Blautia	0	0	0	0	0.0014	0.006
Brevilactibacter	0	0	0	0	0	0
Bulleidia	0.0011	0.0047	0.0019	0.0084	0	0
Campylobacter	0.0395	0.0556	0.0269	0.0548	0.0359	0.0433
Candidatus Saccharimonas	0.0073	0.0254	0.0261	0.0971	0.0021	0.006
Capnocytophaga	0.1133	0.1588	0.0474	0.0874	0.0598	0.0852
Cardiobacterium	0	0	0	0	6e-04	0.0027
Catonella	0.0033	0.0146	0.0152	0.0445	2e-04	8e-04
Cellulomonas	0	0	0.0092	0.0412	0	0
Cellulosilyticum	0	0	0	0	9e-04	0.0039
Centipeda	0	0	0	0	1e-04	4e-04
Christensenella	0	0	0	0	0	0
Clostridium	0	0	0	0	0.001	0.0041
Corynebacterium	0.0024	0.0073	0	0	0.0052	0.0221
Cryptobacterium	0.0061	0.0267	0	0	0	0
Desulfobulbus	0	0	0	0	0	0
Desulfonispora	0	0	0	0	0.0035	0.012
Desulfovibrio	0	0	0	0	0	0

Dialister	0.004	0.0103	0.0017	0.0075	6e-04	0.0018
Dietzia	0	0	0	0	0	0
Dorea	0	0	0	0	0	0
Dysgonomonas	0	0	0.0059	0.0189	0.0032	0.0135
Eggerthia	4e-04	0.0017	0	0	0	0
Eikenella	0	0	0.0049	0.0158	0.024	0.0625
Enterococcus	0.0024	0.0103	0.0117	0.0416	0.0102	0.0307
Erythrobacter	0	0	0	0	0	0
Escherichia	0	0	0	0	0.0012	0.0051
Eubacterium	0.0015	0.0047	9e-04	0.0039	6e-04	0.0019
Faecalicatena	0	0	0	0	0.0017	0.0074
Filifactor	0	0	0	0	9e-04	0.0032
Flavobacterium	0	0	0	0	0	0
Flexilinea	0.0024	0.0105	0	0	0.0032	0.0136
Fretibacterium	0.0025	0.0078	0	0	0.0041	0.0119
Fusibacter	5e-04	0.0022	0	0	0	0
Fusobacterium	0.0359	0.062	0.0421	0.0703	0.03	0.0422
Gemella	0.0138	0.0255	0.01	0.0249	0.0058	0.0137
Gemmobacter	0	0	7e-04	0.0031	0.0025	0.0073
Geosporobacter	0	0	0	0	0.001	0.0044
Granulicatella	0.0165	0.0588	0.0639	0.1434	0.0286	0.089
Haematobacter	0	0	0	0	0	0
Haemophilus	0.0336	0.0647	0.0178	0.0321	0.0099	0.0261
Halomonas	0.0027	0.0118	0	0	0	0
Ihubacter	6e-04	0.0027	0	0	0	0
Johnsonella	0	0	0	0	0.0027	0.0113
Kingella	0.0157	0.0382	0	0	0	0
Kocuria	0	0	0	0	0.0053	0.0225
Lachnoanaerobaculum	0.0037	0.0161	0.0118	0.0448	0.0027	0.0079
Lacrimispora	0	0	0.0011	0.0048	0	0
Lactobacillus	0.0034	0.0147	0.0371	0.1058	0.0683	0.162
Lautropia	0.0023	0.01	0	0	0.0044	0.0187
Lawsonella	0	0	0	0	0	0
Leptotrichia	0.0652	0.0978	0.0123	0.0263	0.0281	0.0336
Mageeibacillus	0	0	0.0032	0.0143	0	0
Megasphaera	0	0	0	0	0.0013	0.0057
Methylobacterium	0	0	0	0	0	0
Microbacterium	0	0	0	0	9e-04	0.0036
Microcella	0	0	0.0013	0.0058	0.0083	0.0335
Micrococcus	0	0	0	0	0	0
Mogibacterium	4e-04	0.0019	0.0034	0.0111	4e-04	0.0017
Mongoliitalea	0	0	0	0	0	0
Moryella	0	0	0	0	0	0
Mycoplasma	0	0	0.0016	0.0073	6e-04	0.0024
Natronohydrobacter	0.0014	0.0061	0	0	0	0
Neisseria	0.0586	0.1054	0.0095	0.0205	0.0066	0.0112
Nesterenkonia	0.0062	0.027	0.0115	0.0394	0.0076	0.024
Nibricoccus	0	0	0	0	0.0059	0.0252
Nitrincola	0	0	0	0	3e-04	0.0014
Okadaella	0	0	0	0	0	0

Olsenella	0.0059	0.0259	0	0	0	0
Oribacterium	0	0	0	0	0	0
Ottowia	0	0	0	0	0.0051	0.0217
Paludibacter	0	0	0.0101	0.0286	0.003	0.0093
Pannonibacter	0	0	0	0	0	0
Parabacteroides	0	0	0.0093	0.0416	5e-04	0.0021
Paracoccus	0	0	0.0031	0.0104	0.0073	0.0155
Pararhodobacter	0	0	0	0	0	0
Parvimonas	0.0019	0.0047	0.0156	0.0697	0.001	0.0023
Peptoanaerobacter	0	0	0	0	0.001	0.0042
Peptococcus	0	0	0.0013	0.0056	0	0
Peptoniphilus	0	0	0	0	5e-04	0.0021
Peptostreptococcus	0.0031	0.0136	0	0	0.0086	0.0221
Phocaeicola	0	0	0.001	0.0045	0	0
Porphyromonas	0.0217	0.035	0.0049	0.0157	0.0281	0.0752
Prevotella	0.0953	0.09	0.0741	0.1117	0.0491	0.0667
Propionibacterium	0	0	0	0	0	0
Prosthecomicrobium	0	0	0	0	0	0
Pseudoleptotrichia	0	0	0	0	5e-04	0.0021
Pseudomonas	0	0	0.0014	0.0063	0.0045	0.0147
Pseudopropionibacterium	0.0063	0.0223	0.0036	0.0161	0.0125	0.0287
Pseudoramibacter	0.0084	0.0367	0	0	0	0
Pseudoruminococcus	0	0	0	0	0	0
Rhodobaca	0.0022	0.0097	0	0	0	0
Rhodobacter	0	0	0	0	6e-04	0.0026
Roseomonas	0	0	0	0	0.0011	0.0045
Rothia	0.0054	0.0142	0.0103	0.0318	0.017	0.0446
Schaalia	0.0035	0.0154	0	0	0.0141	0.0296
Schwartzia	0	0	0	0	0	0
Selenomonas	0.0027	0.0064	0.0052	0.0149	0.0059	0.0098
Shuttleworthia	0.0057	0.0248	0	0	0	0
Slackia	0	0	0.0058	0.0257	0.0049	0.021
Solobacterium	0.0014	0.0056	9e-04	0.0042	0	0
Staphylococcus	0	0	0	0	0	0
Streptococcus	0.1911	0.2047	0.3119	0.3008	0.1796	0.1825
Streptomyces	0	0	0	0	0	0
Tannerella	0.0026	0.008	0.001	0.0045	0.002	0.0048
Tessaracoccus	0	0	0	0	0.0031	0.013
Thermotalea	0	0	0	0	3e-04	0.0013
Treponema	0	0	0.0116	0.0519	0.0124	0.0414
Veillonella	0.0206	0.0313	0.0078	0.015	0.0481	0.1161
Wandonia	0	0	0	0	0	0

	Titanium					
**Genus**	**T0_mean**	**T0_sd**	**T1_mean**	**T1_sd**	**T2_mean**	**T2_sd**
Abiotrophia	0.0127	0.0274	0	0	0.0045	0.0123
Acetobacteroides	0	0	0	0	3e-04	0.0015

Acinetobacter	0	0	0	0	0	0
Actinomyces	0.1207	0.1607	0.292	0.3218	0.3618	0.2683
Aerococcus	0	0	0	0	0	0
Aggregatibacter	0.0034	0.0116	0.0114	0.0346	0.002	0.0086
Alishewanella	0	0	0	0	0	0
Alkalibacterium	0.0275	0.1201	0	0	0.001	0.0041
Alkaliflexus	0	0	0.0066	0.0224	0	0
Alkalihalobacillus	0	0	0	0	0	0
Alkalilacustris	0.0028	0.0123	0	0	0	0
Alkalimonas	0	0	0	0	0	0
Alkaliphilus	0	0	0	0	0	0
Alloprevotella	0.0013	0.0058	0.0024	0.0072	0.0021	0.0091
Aminipila	0	0	0	0	0	0
Anaerobacillus	0	0	0	0	0	0
Anaerobranca	0	0	0	0	0	0
Anaerocella	0	0	0	0	0	0
Anaerocolumna	0	0	0.0048	0.0162	0.0011	0.0048
Anaeroglobus	0	0	0	0	0.0016	0.0039
Anaeromassilibacillus	0.0012	0.0051	0	0	0	0
Anaerovorax	0	0	0	0	4e-04	0.0018
Atopobium	0	0	0.018	0.0785	0.0176	0.065
Azospirillum	0	0	4e-04	0.0018	2e-04	0.001
Bacillus	0	0	0.0142	0.05	0	0
Bacteroides	0	0	0	0	0	0
Belliella	0	0	0	0	0	0
Bergeyella	0.008	0.0272	0.005	0.015	0.0039	0.01
Blautia	0	0	0.0096	0.0289	0	0
Brevilactibacter	0.0106	0.0464	0	0	0	0
Bulleidia	0	0	0	0	0	0
Campylobacter	0.0108	0.0279	0.0159	0.0616	0.0259	0.0318
Candidatus Saccharimonas	4e-04	0.0017	0.0051	0.0199	0.0012	0.0043
Capnocytophaga	0.0986	0.1493	0.0158	0.045	0.0331	0.0549
Cardiobacterium	0	0	0	0	0	0
Catonella	0	0	0	0	0.0017	0.0044
Cellulomonas	0.0028	0.0124	0.0017	0.0074	0	0
Cellulosilyticum	0	0	0	0	0	0
Centipeda	0	0	0	0	0	0
Christensenella	0	0	0	0	0	0
Clostridium	0	0	5e-04	0.0024	0	0
Corynebacterium	0.0064	0.0217	0.002	0.0086	0	0
Cryptobacterium	0	0	0	0	0	0
Desulfobulbus	0	0	0	0	0	0
Desulfonispora	0	0	0	0	2e-04	9e-04
Desulfovibrio	0	0	0	0	3e-04	0.0014
Dialister	0	0	0.0011	0.0049	0.0041	0.0094
Dietzia	0	0	0	0	0	0
Dorea	0	0	0.0159	0.0397	0.0087	0.0287
Dysgonomonas	0	0	0.0029	0.0128	0.002	0.0072
Eggerthia	0	0	0	0	0	0
Eikenella	0	0	0	0	0	0

Enterococcus	0.0025	0.0107	0.0129	0.0418	0.0013	0.0054
Erythrobacter	0	0	0.0079	0.0344	0	0
Escherichia	0	0	0	0	0	0
Eubacterium	0	0	0	0	0.001	0.0023
Faecalicatena	5e-04	0.0021	0.0039	0.017	0	0
Filifactor	0	0	0	0	0.0017	0.0074
Flavobacterium	0	0	0	0	0	0
Flexilinea	0	0	0	0	0.005	0.0211
Fretibacterium	0	0	0.0078	0.0338	0.0061	0.0196
Fusibacter	0	0	0	0	7e-04	0.0021
Fusobacterium	0.0115	0.0158	0.0095	0.0243	0.0199	0.025
Gemella	0.0062	0.0252	0	0	0.0036	0.0097
Gemmobacter	0	0	0.0061	0.0267	9e-04	0.0026
Geosporobacter	0	0	0	0	0	0
Granulicatella	0	0	0.0132	0.0396	0.0299	0.0627
Haematobacter	0	0	0	0	0	0
Haemophilus	0.0432	0.1015	0.0083	0.0285	0.0131	0.0347
Halomonas	0.0049	0.0215	0	0	2e-04	0.001
Ihubacter	0	0	0	0	0	0
Johnsonella	0	0	0	0	0	0
Kingella	0.0628	0.1579	0	0	0	0
Kocuria	0	0	0	0	0	0
Lachnoanaerobaculum	0.0038	0.0165	0.0224	0.0701	0.0053	0.0124
Lacrimispora	0	0	0.001	0.0046	2e-04	0.0011
Lactobacillus	0.0174	0.0648	0.0066	0.0154	0.0214	0.0535
Lautropia	0.0501	0.1838	0.0186	0.0812	0	0
Lawsonella	0	0	0	0	0	0
Leptotrichia	0.0201	0.0481	0.0034	0.0103	0.0148	0.0315
Mageeibacillus	0	0	0	0	0	0
Megasphaera	0.0033	0.0143	0	0	0	0
Methylobacterium	0	0	0	0	0	0
Microbacterium	0.0022	0.0073	0.0063	0.0204	0	0
Microcella	0	0	0	0	0	0
Micrococcus	0	0	0	0	0	0
Mogibacterium	0	0	0.004	0.0176	0	0
Mongoliitalea	0	0	0.0061	0.0268	0	0
Moryella	0.0027	0.0117	0.0139	0.0494	6e-04	0.0026
Mycoplasma	0	0	0	0	0.0015	0.0065
Natronohydrobacter	0.0012	0.0053	0	0	0	0
Neisseria	0.0168	0.0432	0.0075	0.019	0.0192	0.0515
Nesterenkonia	0.0036	0.0156	0	0	0.0024	0.0101
Nibricoccus	0	0	0	0	0	0
Nitrincola	0	0	0	0	0	0
Okadaella	0	0	0.0062	0.0272	4e-04	0.0018
Olsenella	0	0	0	0	0	0
Oribacterium	0	0	9e-04	0.0041	0	0
Ottowia	0	0	0.0093	0.0406	8e-04	0.0036
Paludibacter	0.0025	0.0076	0	0	0.0064	0.0186
Pannonibacter	0	0	0.0014	0.0062	0	0
Parabacteroides	0	0	0.0164	0.0538	8e-04	0.0035

Paracoccus	0	0	0.0059	0.0212	8e-04	0.0033
Pararhodobacter	0	0	0.0054	0.0133	0	0
Parvimonas	4e-04	0.0018	5e-04	0.0024	0.0013	0.0029
Peptoanaerobacter	0	0	0	0	0	0
Peptococcus	0	0	0	0	0	0
Peptoniphilus	0	0	0	0	0	0
Peptostreptococcus	0	0	0	0	0.0063	0.0183
Phocaeicola	0	0	0	0	0	0
Porphyromonas	0.012	0.0295	0.0057	0.0247	0.0248	0.057
Prevotella	0.1113	0.2154	0.0781	0.1215	0.0524	0.0764
Propionibacterium	0	0	5e-04	0.0024	0	0
Prosthecomicrobium	0	0	0.0025	0.0107	0	0
Pseudoleptotrichia	0	0	0	0	0.0029	0.0121
Pseudomonas	0	0	0.0017	0.0073	0.0038	0.0163
Pseudopropionibacterium	0.007	0.0304	0.0033	0.0099	0	0
Pseudoramibacter	0	0	0	0	0.0074	0.0315
Pseudoruminococcus	0	0	0.0041	0.018	0	0
Rhodobaca	0.0027	0.0119	0	0	0	0
Rhodobacter	0	0	0	0	0	0
Roseomonas	0	0	0.0084	0.0214	0	0
Rothia	0	0	0.0172	0.0631	0.01	0.0349
Schaalia	0.0298	0.0709	0	0	0.0196	0.0396
Schwartzia	0	0	0	0	0.0024	0.009
Selenomonas	0.0092	0.0281	0.0078	0.0163	0.0129	0.0183
Shuttleworthia	0	0	0	0	0	0
Slackia	0	0	0	0	0.0028	0.0083
Solobacterium	0	0	0	0	0.0011	0.0038
Staphylococcus	0	0	0	0	0	0
Streptococcus	0.2568	0.2414	0.223	0.2047	0.1713	0.2028
Streptomyces	0	0	0	0	0	0
Tannerella	0.0011	0.005	0	0	0.0026	0.0082
Tessaracoccus	0	0	0	0	0	0
Thermotalea	0	0	0	0	0	0
Treponema	0	0	0.0017	0.0075	0.0087	0.0188
Veillonella	0.0069	0.0134	0.0126	0.0185	0.038	0.0511
Wandonia	0	0	0.0026	0.0115	0	0

	**Tooth**					
**Genus**	**T0_mean**	**T0_sd**	**T1_mean**	**T1_sd**	**T2_mean**	**T2_sd**
Abiotrophia	0	0	0	0	0	0
Acetobacteroides	0	0	0	0	0	0
Acinetobacter	0.014	0.0343	0	0	0	0
Actinomyces	0.2007	0.2538	0.0655	0.061	0.0781	0.1144
Aerococcus	0	0	0	0	0	0
Aggregatibacter	0	0	0	0	0	0
Alishewanella	0	0	0	0	0	0
Alkalibacterium	0	0	0	0	0.0148	0.0266
Alkaliflexus	0	0	0	0	0.0064	0.0099

Alkalihalobacillus	0	0	0	0	0	0
Alkalilacustris	0.0058	0.0143	0	0	0	0
Alkalimonas	0	0	0	0	0	0
Alkaliphilus	0	0	0	0	0	0
Alloprevotella	0.0049	0.0119	0.009	0.0219	0.0021	0.0052
Aminipila	0.007	0.0172	0.0057	0.0089	6e-04	0.0016
Anaerobacillus	0	0	0	0	0	0
Anaerobranca	0	0	0	0	0.0018	0.0043
Anaerocella	0	0	0.0097	0.0238	0.0061	0.015
Anaerocolumna	0	0	0	0	0	0
Anaeroglobus	0	0	0	0	0.0052	0.009
Anaeromassilibacillus	0	0	0	0	0	0
Anaerovorax	0	0	0	0	0	0
Atopobium	0	0	0	0	0.0098	0.016
Azospirillum	0	0	0	0	0	0
Bacillus	0	0	0	0	0.0214	0.0419
Bacteroides	0	0	0.0015	0.0037	0.0017	0.0041
Belliella	0	0	0	0	0.007	0.0171
Bergeyella	0	0	0	0	0	0
Blautia	0	0	0	0	0.0088	0.0138
Brevilactibacter	0	0	0	0	0	0
Bulleidia	9e-04	0.0021	0	0	0	0
Campylobacter	0.0313	0.0491	0.0246	0.0475	0.0182	0.0293
Candidatus Saccharimonas	0.0049	0.0121	0.014	0.022	0.0099	0.0241
Capnocytophaga	0.0132	0.0323	0.0343	0.0696	0	0
Cardiobacterium	0	0	0	0	0	0
Catonella	0	0	0	0	0	0
Cellulomonas	0	0	0	0	0	0
Cellulosilyticum	0	0	0	0	0	0
Centipeda	0	0	0	0	0	0
Christensenella	0.0034	0.0083	0.0016	0.0039	0	0
Clostridium	0	0	0	0	0	0
Corynebacterium	0.0127	0.031	0.0305	0.0616	0.0138	0.0155
Cryptobacterium	0	0	0	0	0	0
Desulfobulbus	0	0	0.0047	0.0116	0	0
Desulfonispora	0	0	0	0	0.0025	0.0041
Desulfovibrio	0	0	0	0	0	0
Dialister	0	0	0	0	0.0022	0.0035
Dietzia	0	0	0.0146	0.0358	0	0
Dorea	0	0	0	0	0.0674	0.0992
Dysgonomonas	0	0	0	0	0.0085	0.0208
Eggerthia	0	0	0	0	0	0
Eikenella	0.0153	0.0374	0	0	0	0
Enterococcus	0	0	0	0	0.027	0.0281
Erythrobacter	0	0	0	0	0	0
Escherichia	0	0	0	0	0	0
Eubacterium	0.0059	0.0122	0.0037	0.009	0.0049	0.0072
Faecalicatena	0	0	0	0	0.0045	0.007
Filifactor	0	0	0	0	0	0
Flavobacterium	0	0	0	0	0.0032	0.0078

Flexilinea	0.0037	0.0091	0	0	0	0
Fretibacterium	0.0224	0.0391	0.0064	0.0156	0.0021	0.0053
Fusibacter	0.004	0.0063	0.005	0.0083	0	0
Fusobacterium	0.0518	0.0694	0.0595	0.0571	0.0243	0.0251
Gemella	0.0028	0.0069	0	0	0	0
Gemmobacter	0	0	0	0	0.0052	0.0128
Geosporobacter	0	0	0	0	0	0
Granulicatella	0	0	0	0	0	0
Haematobacter	0.0032	0.0078	0	0	0	0
Haemophilus	0.0275	0.0378	0	0	0.0035	0.0055
Halomonas	0	0	0	0	0	0
Ihubacter	0	0	0	0	0	0
Johnsonella	0	0	0	0	0	0
Kingella	0	0	0	0	0	0
Kocuria	0.0098	0.024	0	0	0.0065	0.016
Lachnoanaerobaculum	0	0	0	0	0	0
Lacrimispora	0	0	0	0	0	0
Lactobacillus	0.0087	0.0212	0	0	0	0
Lautropia	0.013	0.0318	0	0	0.0091	0.0222
Lawsonella	0	0	0	0	8e-04	0.002
Leptotrichia	0.0207	0.0414	0.0505	0.0999	0	0
Mageeibacillus	0	0	0	0	0	0
Megasphaera	0.0087	0.0213	0	0	0	0
Methylobacterium	0	0	0	0	0	0
Microbacterium	0	0	0.0032	0.0078	0.0041	0.0101
Microcella	0	0	0	0	0.0044	0.0108
Micrococcus	0	0	0.016	0.0392	0	0
Mogibacterium	0	0	0.0047	0.0116	0.0017	0.0041
Mongoliitalea	0	0	0	0	0.0062	0.0152
Moryella	0	0	0	0	0	0
Mycoplasma	0.0026	0.0064	0	0	0.0059	0.0144
Natronohydrobacter	0	0	0	0	0	0
Neisseria	0.0093	0.0228	0.004	0.0099	0.0015	0.0037
Nesterenkonia	0	0	0.021	0.0513	0.0017	0.0041
Nibricoccus	0	0	0	0	0.0206	0.0505
Nitrincola	0	0	0	0	0	0
Okadaella	0	0	0	0	0	0
Olsenella	0	0	0	0	0.018	0.0395
Oribacterium	0	0	0	0	0	0
Ottowia	0	0	0	0	0	0
Paludibacter	0.0112	0.0207	0	0	6e-04	0.0016
Pannonibacter	0	0	0	0	0	0
Parabacteroides	0	0	0	0	0.0103	0.0252
Paracoccus	0	0	0	0	0.0075	0.0137
Pararhodobacter	0	0	0	0	0.0023	0.0056
Parvimonas	0	0	0.0065	0.016	0.0023	0.0039
Peptoanaerobacter	0	0	0	0	0	0
Peptococcus	0	0	0	0	0	0
Peptoniphilus	0	0	0	0	0	0
Peptostreptococcus	0	0	0.1015	0.1613	0.0202	0.0494

Phocaeicola	0	0	0	0	0.0029	0.007
Porphyromonas	0.0682	0.167	0.0154	0.0337	0.0101	0.0226
Prevotella	0.0786	0.1198	0.129	0.1747	0.0814	0.1038
Propionibacterium	0	0	0.012	0.0295	0.0011	0.0028
Prosthecomicrobium	0	0	0	0	0	0
Pseudoleptotrichia	0	0	0	0	0	0
Pseudomonas	0	0	0	0	0.0116	0.0152
Pseudopropionibacterium	0	0	0	0	0	0
Pseudoramibacter	0	0	0	0	0.0711	0.1103
Pseudoruminococcus	0	0	0	0	0	0
Rhodobaca	0	0	0	0	0	0
Rhodobacter	0	0	0	0	0.0017	0.0041
Roseomonas	0	0	0	0	0.0094	0.0104
Rothia	0.0044	0.0108	0	0	0	0
Schaalia	0.0317	0.05	0	0	0	0
Schwartzia	0	0	0	0	0	0
Selenomonas	8e-04	0.002	0.0248	0.0536	0.0025	0.0061
Shuttleworthia	0	0	0	0	0	0
Slackia	0.01	0.0244	0.0246	0.0415	0.0276	0.0676
Solobacterium	0	0	0.0042	0.0102	8e-04	0.0019
Staphylococcus	0	0	0	0	0	0
Streptococcus	0.0936	0.0953	0.2778	0.3683	0.2885	0.3954
Streptomyces	0	0	0	0	0	0
Tannerella	0	0	0	0	0	0
Tessaracoccus	0	0	0	0	0	0
Thermotalea	0	0	0	0	0	0
Treponema	0.0156	0.0254	0.0091	0.0222	0	0
Veillonella	0.1777	0.3827	0.0056	0.0107	0.001	0.0019
Wandonia	0	0	0	0	0.006	0.0147
