# Supplementary material for: Microbial evaluation of zirconia and titanium implants in the anterior mandibula: a randomized controlled clinical trial
Source: Sci Rep. 2026 Jun 3;16:17031. doi: 10.1038/s41598-026-54915-0 (PMC13230837; doi:10.1038/s41598-026-54915-0)
Supplement: Supplementary file 3 — Supplementary Material 3 [file 41598_2026_54915_MOESM3_ESM.pdf]

	Zirconium					
**Species**	**T0_mean**	**T0_sd**	**T1_mean**	**T1_sd**	**T2_mean**	**T2_sd**
Abiotrophia defectiva	0.0117	0.0393	0.0108	0.0483	0.0102	0.0409
Acetobacteroides hydrogenigenes	0	0	0	0	0	0
Acinetobacter sp. G3DM-29	0	0	0	0	0	0
Actinomyces dentalis	0	0	0	0	0	0
Actinomyces genomosp. C1	0	0	0	0	0	0
Actinomyces gerencseriae	0	0	0	0	0	0
Actinomyces graevenitzii	0	0	0.0086	0.0386	0	0
Actinomyces israelii	0	0	0.0144	0.0642	0.0258	0.0978
Actinomyces naeslundii	0	0	0	0	0.0106	0.0451
Actinomyces oris	0.0103	0.045	0	0	0.0224	0.0465
Actinomyces provencensis	0	0	0.003	0.0132	0.0042	0.0178
Actinomyces sp.	0	0	0	0	0	0
Actinomyces sp. 'ARUP UnID 105'	0	0	0	0	0.0026	0.0112
Actinomyces sp. 'ARUP UnID 46'	0.0045	0.0195	0.0049	0.0218	0.0093	0.0305
Actinomyces sp. 'ARUP UnID 51'	0	0	0	0	0.0033	0.0141
Actinomyces sp. 'ARUP UnID 53'	0	0	0	0	0.0042	0.0177
Actinomyces sp. 'ARUP UnID 56'	0	0	0	0	0	0
Actinomyces sp. 'ARUP UnID 58'	0	0	0	0	0	0
Actinomyces sp. 'ARUP UnID 59'	0	0	0	0	0	0
Actinomyces sp. 'ARUP UnID 62'	0.0062	0.0272	0.0054	0.024	0	0
Actinomyces sp. 'ARUP UnID 70'	0.0299	0.1302	0	0	0.0049	0.0206
Actinomyces sp. 'ARUP UnID 71'	0	0	0	0	0	0
Actinomyces sp. 'ARUP UnID 72'	0.0242	0.1054	0	0	0	0
Actinomyces sp. 'ARUP UnID 77'	0	0	0	0	0	0
Actinomyces sp. 'ARUP UnID 89'	0	0	0.0048	0.0215	0.0258	0.0894
Actinomyces sp. 'ARUP UnID 95'	0	0	0	0	0	0
Actinomyces sp. 11-179	0	0	0	0	0.0065	0.0276
Actinomyces sp. 12-664	0	0	0.0054	0.0243	0.0053	0.0226
Actinomyces sp. 13-266	0	0	0	0	0.0067	0.0285
Actinomyces sp. A1	0	0	0	0	0.0012	0.005
Actinomyces sp. A3	0	0	0	0	0.0032	0.0097
Actinomyces sp. ChDC B197	0	0	0	0	0	0
Actinomyces sp. ChDC B642	0	0	0.0092	0.0414	0	0
Actinomyces sp. ChDC B645	0	0	0.0222	0.0992	0.0067	0.0284
Actinomyces sp. ICM39	0	0	0	0	0.0027	0.0114
Actinomyces sp. oral strain Hal-1065	0	0	0.0073	0.0325	0	0
Actinomyces sp. oral taxon 169	0	0	0	0	0.0037	0.0156
Actinomyces sp. oral taxon 170	0	0	0	0	0.0072	0.0223
Actinomyces sp. oral taxon 171	0	0	0	0	0	0
Actinomyces sp. oral taxon 175	0	0	0	0	0.0068	0.0289
Actinomyces sp. oral taxon 525	0	0	0.017	0.0761	0	0
Actinomyces sp. oral taxon 897	0	0	0.0103	0.0316	0.0088	0.0281
Actinomyces sp. oral taxon A50	0.0055	0.0241	0.008	0.0358	0.0054	0.023
Actinomyces sp. oral taxon B78	0	0	0	0	0	0
Actinomyces sp. oral taxon E63	0	0	0	0	0	0
Actinomyces sp. R42.11	0	0	0.0138	0.0616	0	0

Actinomyces sp. R42.5	0	0	0	0	0	0
Actinomyces viscosus	0	0	0.0095	0.0425	0	0
Aerococcus christensenii	0	0	0	0	0.0012	0.0052
Aggregatibacter aphrophilus	0	0	0	0	0.0014	0.006
Aggregatibacter kilianii	0	0	0	0	6e-04	0.0027
Aggregatibacter segnis	0	0	0.0064	0.0216	0.004	0.0119
Aggregatibacter sp. 316364/07	0.0012	0.0051	0	0	0	0
Aggregatibacter sp. HS19_2W_I12	0.0023	0.01	0	0	0	0
Alishewanella alkalitolerans	0	0	0	0	0.0023	0.0096
Alkaliflexus imshenetskii	0	0	0	0	0.0024	0.0101
Alkalihalobacillus alkalisediminis	0	0	0	0	4e-04	0.0019
Alkalilacustris brevis	0.0102	0.0351	0.0012	0.0055	0	0
Alkalimonas sp. BW86-79	0	0	0	0	0.0065	0.0274
Alkaliphilus hydrothermalis	0	0	0	0	4e-04	0.0017
Alkaliphilus sp.	0	0	0	0	8e-04	0.0023
Alloprevotella Prevotella sp. oral taxon 308	0.0011	0.0046	0	0	0	0
Alloprevotella rava	0.0023	0.01	0	0	0	0
Alloprevotella tannerae	0.0147	0.0484	0	0	0	0
Anaerobacillus sp.	0	0	0	0	8e-04	0.0035
Anaerobacillus sp. M5-13	0	0	0	0	0.0018	0.0059
Anaerobranca sp. S55_26_2	0	0	0	0	7e-04	0.0028
Anaerocella delicata	0	0	0	0	0	0
Anaerocolumna cellulosilytica	0	0	0	0	0	0
Anaerocolumna xylanovorans	0	0	0	0	0	0
Anaeroglobus geminatus	0.002	0.0085	0	0	0.0023	0.0065
Anaeromassilibacillus sp. Marseille-P3371	0	0	0	0	0	0
Anaerovorax odorimutans	0	0	0	0	0	0
Atopobium deltae	0	0	0	0	0.001	0.0044
Atopobium parvulum	0.0059	0.0256	0	0	0.0093	0.0395
Atopobium rimae	0	0	0.0049	0.0221	0	0
Atopobium sp. DMCT15023	0	0	0	0	0	0
Atopobium vaginae	0	0	0	0	0	0
Azospirillum sp. AP-500	0	0	0	0	0	0
Bacillus cellulosilyticus	0	0	0	0	0.0044	0.0137
Bacillus sp. ANL-isoa2	0	0	6e-04	0.0028	0.0029	0.0091
Bacillus sp. oral taxon C44	0.0045	0.0198	8e-04	0.0038	0.0125	0.0387
Bacteroides heparinolyticus	0	0	0.0056	0.0253	0.0031	0.0131
Bacteroides salyersiae	0	0	0	0	0	0
Belliella sp. LW3	0	0	0	0	0	0
Bergeyella sp. AF14	0.0049	0.0215	0.0121	0.045	0.0067	0.0165
Bergeyella sp. oral taxon 322	0	0	0.0022	0.0098	6e-04	0.0025
Blautia sp. Marseille-P3313	0	0	0	0	0.0021	0.0091
Brevilactibacter sinopodophylli	0	0	0	0	0	0
Bulleidia extructa	0.0012	0.0052	0.0021	0.0094	0	0
Campylobacter concisus	0.0013	0.0055	0	0	0	0
Campylobacter curvus	0.0187	0.0662	0.0025	0.0111	0.005	0.0151
Campylobacter gracilis	0.0032	0.0138	0.0116	0.0517	0.0188	0.0267
Campylobacter rectus	0	0	0	0	0.0045	0.019

Campylobacter showae	0.0052	0.0228	0.01	0.0351	0.0078	0.0292
Campylobacter sp. FOBRC14	0	0	0.0038	0.017	0	0
Campylobacter sp. oral taxon G43	0	0	0	0	0	0
Candidatus Saccharimonas aalborgensis	0	0	0	0	0	0
Candidatus Saccharimonas sp.	0.0076	0.0262	0.0291	0.1049	0.0024	0.007
Capnocytophaga genosp. AHN8471	0	0	0	0	0.0035	0.0102
Capnocytophaga gingivalis	0.0101	0.0278	0.0119	0.0421	0.0212	0.0596
Capnocytophaga granulosa	0	0	0.0051	0.0188	0.0084	0.031
Capnocytophaga leadbetteri	0.0122	0.0329	0.0139	0.0534	0.0034	0.0085
Capnocytophaga ochracea	0	0	0	0	0.0067	0.0284
Capnocytophaga sp.	0.0018	0.0078	0	0	0.0013	0.0056
Capnocytophaga sp. 'ARUP UnID 181'	0	0	0	0	0	0
Capnocytophaga sp. 'ARUP UnID 182'	0	0	0	0	0.0022	0.0093
Capnocytophaga sp. 'ARUP UnID 184'	0	0	0	0	0.0034	0.0144
Capnocytophaga sp. AHN10044	0	0	0	0	0.0037	0.0157
Capnocytophaga sp. AHN9756	0	0	0.0065	0.0293	0	0
Capnocytophaga sp. FDAARGOS_737	0.0221	0.0756	0.0018	0.008	0.0064	0.0227
Capnocytophaga sp. FVAMC 7623	0	0	0	0	0.0033	0.014
Capnocytophaga sp. oral taxon 336	0	0	0	0	0	0
Capnocytophaga sp. oral taxon 338	0	0	0	0	0	0
Capnocytophaga sp. oral taxon 864	0	0	0	0	0	0
Capnocytophaga sp. oral taxon A48	0.0079	0.0342	0	0	0	0
Capnocytophaga sp. oral taxon B29	0	0	0	0	6e-04	0.0027
Capnocytophaga sputigena	0.078	0.1647	0.0163	0.0503	0.0054	0.0159
Cardiobacterium sp. 'ARUP UnID 187'	0	0	0	0	8e-04	0.0032
Catonella morbi	0.0037	0.0161	0.0173	0.0499	2e-04	9e-04
Catonella sp. oral taxon F17	0	0	0	0	0	0
Cellulomonas hominis	0	0	0.0125	0.0559	0	0
Centipeda sp. oral taxon F73	0	0	0	0	1e-04	5e-04
Christensenella minuta	0	0	0	0	0	0
Clostridium sp. 6-44	0	0	0	0	0.0013	0.0054
Corynebacterium durum	0	0	0	0	0	0
Corynebacterium matruchotii	0.0013	0.0057	0	0	8e-04	0.0035
Corynebacterium sp. oral taxon B00	0	0	0	0	0	0
Corynebacterium sp. ZT10-3	0	0	0	0	0	0
Cryptobacterium curtum	0.0072	0.0314	0	0	0	0
Desulfobulbus oralis	0	0	0	0	0	0
Desulfonispora thiosulfatigenes	0	0	0	0	0.0048	0.016
Desulfovibrio sp. feline oral taxon 347	0	0	0	0	0	0
Dialister invisus	0.0039	0.0096	0.0019	0.0084	4e-04	0.0013
Dialister micraerophilus	0	0	0	0	0	0
Dialister pneumosintes	7e-04	0.003	0	0	0	0
Dialister sp. E2_20	0	0	0	0	0	0
Dialister sp. oral taxon C66	0	0	0	0	0	0
Dorea Candidatus Dorea massiliensis	0	0	0	0	0	0
Dysgonomonas sp. Marseille-P4677	0	0	0.0532	0.2233	0.0049	0.0206
Eggerthia catenaformis	5e-04	0.002	0	0	0	0
Eikenella corrodens	0	0	0.0057	0.0182	0.0238	0.0582
Eikenella longinqua	0	0	0	0	0.0032	0.0137

Enterococcus italicus	0	0	0	0	0.0013	0.0053
Enterococcus sp. oral taxon A43	0	0	0	0	0.0046	0.0139
Erythrobacter sp. MED13	0	0	0	0	0	0
Escherichia coli	0	0	0	0	0.0025	0.0105
Eubacterium sp.	0	0	0.0011	0.005	3e-04	0.0012
Eubacterium sp. oral strain A35MT	0.0017	0.0055	0	0	0	0
Eubacterium sp. oral taxon G32	0	0	0	0	6e-04	0.0025
Faecalicatena fissicatena	0	0	0	0	0.0023	0.0098
Filifactor alocis	0	0	0	0	0.0012	0.0039
Flavobacterium sp.	0	0	0	0	0	0
Flexilinea flocculi	0.0027	0.0117	0	0	0.005	0.0212
Fretibacterium fastidiosum	0.001	0.0042	0	0	0.0017	0.0071
Fretibacterium sp. feline oral taxon 223	0.0018	0.0056	0	0	0.0039	0.0113
Fusibacter paucivorans	6e-04	0.0025	0	0	0	0
Fusobacterium hwasookii	0.0021	0.0072	7e-04	0.0031	0	0
Fusobacterium naviforme	0	0	0	0	0	0
Fusobacterium nucleatum	0.043	0.1009	0.0247	0.0523	0.0324	0.0496
Fusobacterium pseudoperiodonticum	0.0023	0.007	7e-04	0.0032	3e-04	0.0013
Fusobacterium sp. oral taxon 203	0.003	0.0131	0.0116	0.0518	0.0019	0.0081
Gemella haemolysans	0.0208	0.0403	0.0128	0.0323	0.0032	0.0138
Gemella morbillorum	0	0	0	0	0.0011	0.0032
Gemmobacter sp. YIM 102744-1	0	0	0.0011	0.0049	0.0033	0.0098
Geosporobacter ferrireducens	0	0	0	0	0.0014	0.0058
Granulicatella adiacens	0.0037	0.0162	0.0672	0.1577	0.0442	0.1444
Granulicatella elegans	0.0159	0.0692	0.0089	0.0399	0	0
Haematobacter massiliensis	0	0	0	0	0	0
Haemophilus haemolyticus	0.0032	0.0139	0.0066	0.0294	0	0
Haemophilus influenzae	0	0	0	0	9e-04	0.0038
Haemophilus parahaemolyticus	0	0	0	0	0	0
Haemophilus parainfluenzae	0.0332	0.0823	0.0114	0.0295	0.0054	0.0173
Haemophilus paraphrohaemolyticus	0	0	0	0	0.0152	0.0644
Haemophilus sp. CCUG 13929	0.0037	0.016	0.0017	0.0074	0	0
Haemophilus sp. CCUG 23622	0	0	0	0	9e-04	0.0039
Haemophilus sp. oral taxon 036	0.002	0.0085	0	0	0	0
Haemophilus sputorum	0	0	6e-04	0.0028	0	0
Ihubacter massiliensis	7e-04	0.003	0	0	0	0
Johnsonella ignava	0	0	0	0	0.003	0.0125
Kingella denitrificans	0.0212	0.052	0	0	0	0
Kingella oralis	0	0	0	0	0	0
Lachnoanaerobaculum cf. saburreum oral strain C27KA	0	0	0	0	0.0016	0.0067
Lachnoanaerobaculum gingivalis	0.005	0.0218	0	0	6e-04	0.0026
Lachnoanaerobaculum orale	0	0	0.0056	0.025	0	0
Lachnoanaerobaculum saburreum	0	0	0.0017	0.0076	0	0
Lacrimispora saccharolytica	0	0	0.0012	0.0053	0	0
Lactobacillus delbrueckii	0	0	0	0	0	0
Lactobacillus fermentum	0	0	0	0	0	0
Lactobacillus gasseri	0	0	0	0	0.0013	0.0055
Lactobacillus kitasatonis	0	0	0	0	0	0
Lactobacillus paracasei	0	0	0	0	0.0045	0.0193

Lactobacillus reuteri	0	0	0	0	0.0013	0.0057
Lactobacillus rhamnosus	0	0	0	0	7e-04	0.003
Lactobacillus salivarius	0.0011	0.005	6e-04	0.0026	0	0
Lactobacillus sp. 46-211	0	0	0	0	9e-04	0.0039
Lactobacillus vaginalis	0	0	0.0168	0.075	0.0033	0.0095
Lautropia mirabilis	0.0045	0.0198	0	0	0.0053	0.0226
Lawsonella clevelandensis	0	0	0	0	0	0
Leptotrichia buccalis	0.0046	0.0184	8e-04	0.0037	0	0
Leptotrichia genomosp. C1	0.0015	0.0049	0	0	0.0019	0.0082
Leptotrichia massiliensis	0	0	0	0	2e-04	8e-04
Leptotrichia shahii	0	0	3e-04	0.0015	2e-04	7e-04
Leptotrichia sp.	0.0057	0.0247	0	0	0.0081	0.0161
Leptotrichia sp. oral taxon 212	0.023	0.0886	0	0	2e-04	0.001
Leptotrichia sp. oral taxon 215	0.0052	0.0147	0.0046	0.0207	0.0019	0.0056
Leptotrichia sp. oral taxon 217	0.0065	0.0208	0.0025	0.0111	0.0025	0.0106
Leptotrichia sp. oral taxon 218	0.012	0.0523	0	0	0	0
Leptotrichia sp. oral taxon 219	0	0	8e-04	0.0034	7e-04	0.0021
Leptotrichia sp. oral taxon 221	0.0031	0.0134	5e-04	0.0024	0	0
Leptotrichia sp. oral taxon 225	0.0026	0.0098	5e-04	0.0024	8e-04	0.0025
Leptotrichia sp. oral taxon 392	0	0	0	0	0	0
Leptotrichia sp. oral taxon 463	6e-04	0.0028	0	0	0	0
Leptotrichia sp. oral taxon 498	0.0042	0.0184	0	0	0	0
Leptotrichia trevisanii	0.0121	0.051	0.0013	0.004	0.0035	0.0132
Leptotrichia wadei	0.0104	0.0315	0.0035	0.0104	0.0188	0.026
Mageeibacillus indolicus	0	0	0.0044	0.0195	0	0
Megasphaera genomosp. C1	0	0	0	0	0.0013	0.0054
Megasphaera micronuciformis	0	0	0	0	7e-04	0.0029
Methylobacterium haplocladii	0	0	0	0	0	0
Microbacterium sp. NDK-63	0	0	0	0	0	0
Microcella alkaliphila	0	0	0.002	0.0091	0.0127	0.051
Mogibacterium timidum	5e-04	0.0021	0.0013	0.0056	6e-04	0.0026
Mongoliitalea sp.	0	0	0	0	0	0
Moryella sp. KHD1	0	0	0	0	0	0
Mycoplasma faucium	0	0	0	0	0	0
Mycoplasma hyorhinis	0	0	0.0021	0.0094	0	0
Mycoplasma salivarium	0	0	0	0	6e-04	0.0025
Natronohydrobacter thiooxidans	0.0032	0.0141	0	0	0	0
Neisseria bacilliformis	0	0	0	0	0	0
Neisseria elongata	0	0	0	0	0.0019	0.0058
Neisseria flava	0.0023	0.0101	0	0	0	0
Neisseria flavescens	0	0	0	0	0	0
Neisseria sp. 'ARUP UnID 432'	0.0046	0.0202	0	0	0	0
Neisseria sp. oral taxon 014	0	0	0	0	0	0
Neisseria sp. oral taxon 499	0	0	0	0	0	0
Neisseria subflava	0.006	0.0261	0.0045	0.0202	0.0025	0.0107
Nesterenkonia sp.	0	0	0.0162	0.0544	0.0056	0.0178
Nibricoccus aquaticus	0	0	0	0	0.0079	0.0335
Nitrincola sp. E-044	0	0	0	0	5e-04	0.0022
Okadaella gastrococcus	0	0	0	0	0	0
Olsenella sp. F0004	0	0	0	0	0	0

Oribacterium sp. oral taxon 078	0	0	0	0	0	0
Paludibacter propionicigenes	0	0	0.0123	0.0343	0.0042	0.0138
Parabacteroides sp. Marseille-P3160	0	0	0.01	0.0447	7e-04	0.0029
Paracoccus sp.	0	0	0	0	0.0026	0.0112
Pararhodobacter sp.	0	0	0	0	0	0
Parvimonas micra	0.0014	0.0048	0.0168	0.0749	0.0013	0.003
Parvimonas sp. canine oral taxon 102	7e-04	0.003	0	0	0	0
Peptoanaerobacter [Eubacterium] yurii	0	0	0	0	0.0012	0.005
Peptococcus sp. oral taxon 168	0	0	0.0016	0.0072	0	0
Peptoniphilus sp. oral taxon 836	0	0	0	0	7e-04	0.0029
Peptostreptococcus sp. CCUG 42997	0.0072	0.0314	0	0	0.0038	0.0162
Peptostreptococcus sp. oral taxon 113	0	0	0	0	0.0051	0.0216
Peptostreptococcus stomatis	0	0	0	0	0.0017	0.0071
Phocaeicola abscessus	0	0	0.0013	0.0057	0	0
Porphyromonas catoniae	0.0026	0.0112	0	0	0.0038	0.0162
Porphyromonas endodontalis	0.004	0.0176	0	0	0.01	0.0275
Porphyromonas gingivalis	0.003	0.0129	0.0021	0.0094	0.0173	0.0576
Porphyromonas pasteri	0.0147	0.0404	0.0047	0.0212	0	0
Porphyromonas sp. oral taxon 275	0.0021	0.009	0	0	0	0
Porphyromonas sp. oral taxon 278	0.0012	0.0054	0	0	0	0
Prevotella baroniae	0	0	0	0	0	0
Prevotella conceptionensis	0	0	0	0	0	0
Prevotella dentalis	0	0	0	0	0	0
Prevotella denticola	0	0	0	0	0	0
Prevotella genomosp. C1	0	0	0.0024	0.0108	0	0
Prevotella genomosp. P6	0	0	0	0	0	0
Prevotella intermedia	0	0	0	0	0.0026	0.011
Prevotella koreensis	0.0011	0.0046	0	0	0	0
Prevotella loescheii	0	0	0.0055	0.0248	7e-04	0.0023
Prevotella maculosa	0.0043	0.0186	0	0	7e-04	0.003
Prevotella melaninogenica	0.0374	0.0613	0.0475	0.1268	0.0136	0.0467
Prevotella micans	0.0024	0.0103	0	0	0	0
Prevotella multiformis	0.0099	0.0433	0	0	0	0
Prevotella nanceiensis	0	0	0.0071	0.0317	0	0
Prevotella oralis	0	0	0	0	0	0
Prevotella oris	0.0205	0.0621	0.0104	0.0374	0.0291	0.0735
Prevotella oulorum	0.0078	0.0249	0.0056	0.0176	8e-04	0.0036
Prevotella pallens	0	0	0	0	0	0
Prevotella salivae	0.0093	0.0242	0.0021	0.0095	0.0058	0.0175
Prevotella sp. 8404125	0	0	0.0029	0.0129	0	0
Prevotella sp. CM38	0	0	0	0	0	0
Prevotella sp. oral taxon 292	0	0	0	0	0	0
Prevotella sp. oral taxon 300	0.0088	0.0382	0	0	0	0
Prevotella sp. oral taxon 303	0	0	0	0	0	0
Prevotella sp. oral taxon 314	0.0017	0.0073	0	0	0.0096	0.0291
Prevotella sp. oral taxon 317	0	0	0.0027	0.0121	0.0051	0.0214
Prevotella sp. oral taxon 472	0.0044	0.0191	0.0044	0.0196	0.0016	0.0066
Prevotella sp. oral taxon 475	0.0023	0.0099	0.0037	0.0163	0.0011	0.0046
Prevotella sp. oral taxon 515	0	0	0	0	0	0
Prevotella sp. oral taxon G60	0	0	0	0	0	0

Prosthecomicrobium sp. 'ARUP UnID 533'	0	0	0	0	0	0
Pseudoleptotrichia goodfellowii	0	0	0	0	6e-04	0.0027
Pseudomonas tarimensis	0	0	0	0	5e-04	0.0022
Pseudomonas xinjiangensis	0	0	0.0022	0.0098	0.006	0.0222
Pseudopropionibacterium massiliense	0	0	0	0	0.0111	0.0326
Pseudopropionibacterium propionicum	0.0119	0.0458	0.0049	0.0218	0.0034	0.0116
Pseudoramibacter alactolyticus	0.0093	0.0406	0	0	0	0
Pseudoruminococcus massiliensis	0	0	0	0	0	0
Rhodobacter sp. CCP-1	0	0	0	0	9e-04	0.004
Roseomonas sp.	0	0	0	0	0.0016	0.0069
Rothia dentocariosa	0	0	0	0	0	0
Rothia mucilaginosa	0.001	0.0043	0	0	8e-04	0.0035
Schaalia cardiffensis	0.0039	0.017	0	0	0	0
Schaalia georgiae	0	0	0	0	0.008	0.0248
Schaalia meyeri	0	0	0	0	0.0022	0.0095
Schaalia odontolytica	0	0	0	0	0.0089	0.0377
Schwartzia sp. canine oral taxon 042	0	0	0	0	0	0
Selenomonas artemidis	0	0	0	0	0.0013	0.0054
Selenomonas dianae	0	0	0.0011	0.0051	0	0
Selenomonas infelix	0	0	0.0032	0.0144	0.0016	0.0066
Selenomonas noxia	0.0015	0.0064	0	0	0.0017	0.0071
Selenomonas sp. oral taxon 126	0	0	0	0	1e-04	4e-04
Selenomonas sp. oral taxon 134	0	0	0	0	0	0
Selenomonas sp. oral taxon 137	7e-04	0.0031	0	0	0	0
Selenomonas sp. oral taxon 138	0	0	0	0	0	0
Selenomonas sp. oral taxon 149	4e-04	0.0017	0	0	0	0
Selenomonas sp. oral taxon 920	0	0	7e-04	0.0033	4e-04	0.0017
Selenomonas sp. oral taxon F19	0	0	0	0	0	0
Selenomonas sp. oral taxon F20	0	0	0	0	0	0
Selenomonas sp. oral taxon F21	0	0	0	0	0	0
Selenomonas sp. oral taxon F72	2e-04	0.0011	0	0	0	0
Selenomonas sp. oral taxon F81	0	0	0	0	0	0
Selenomonas sp. oral taxon F82	5e-04	0.0024	0	0	8e-04	0.0029
Selenomonas sp. oral taxon G00	0	0	0	0	0	0
Selenomonas sp. oral taxon G67	0	0	0	0	0	0
Selenomonas sp. oral taxon H64	0	0	0	0	0	0
Selenomonas sp. oral taxon H65	0	0	2e-04	9e-04	0	0
Selenomonas sp. oral taxon H66	0	0	0	0	1e-04	5e-04
Selenomonas sputigena	0	0	0	0	0	0
Shuttleworthia satelles	0.0067	0.0292	0	0	0	0
Slackia exigua	0	0	0.0064	0.0288	0.0067	0.0285
Solobacterium moorei	0.0016	0.0062	0.0012	0.0054	0	0
Staphylococcus aureus	0	0	0	0	0	0
Streptococcus anginosus	0	0	0.0461	0.206	0.0063	0.0265
Streptococcus australis	0.0024	0.0105	0.0053	0.0169	3e-04	0.0012
Streptococcus constellatus	0	0	0	0	2e-04	0.001
Streptococcus cristatus	0	0	0	0	0	0
Streptococcus downei	0.0013	0.0055	0	0	0.0054	0.023
Streptococcus gordonii	6e-04	0.0027	0.0201	0.0898	0.025	0.0797

Streptococcus gwangjuense	0	0	0	0	0	0
Streptococcus infantis	0.0169	0.0735	0.0021	0.0095	0.0025	0.0107
Streptococcus intermedius	0	0	0	0	1e-04	5e-04
Streptococcus mitis	0.0597	0.1276	0.0706	0.21	0.0057	0.0138
Streptococcus mutans	0	0	8e-04	0.0038	0.0123	0.0522
Streptococcus oralis	0.0023	0.01	0	0	0.0057	0.0242
Streptococcus parasanguinis	0.0245	0.0618	0.0028	0.0126	0.0123	0.0234
Streptococcus periodonticum	0	0	0	0	0.0013	0.0053
Streptococcus peroris	0	0	0	0	0.0014	0.006
Streptococcus pneumoniae	0.0011	0.0048	0	0	0	0
Streptococcus salivarius	0	0	0	0	0.0012	0.005
Streptococcus sanguinis	0.0215	0.0415	0.0236	0.0602	0.0185	0.0563
Streptococcus sp.	0.0014	0.006	9e-04	0.0041	0	0
Streptococcus sp. 'ARUP UnID 614'	0	0	0	0	0	0
Streptococcus sp. 'ARUP UnID 639'	0	0	0	0	3e-04	0.0012
Streptococcus sp. 10aMclG2	0.011	0.0427	0	0	0	0
Streptococcus sp. 10aVMg2	0	0	0.0015	0.0069	6e-04	0.0024
Streptococcus sp. 13aVMg2	0	0	0	0	0	0
Streptococcus sp. A12	0	0	0	0	0	0
Streptococcus sp. AS14	0	0	0.0022	0.0098	0.0033	0.0139
Streptococcus sp. B2	0.0014	0.0061	0	0	0	0
Streptococcus sp. ChDC B366	0	0	0	0	0	0
Streptococcus sp. ChDC B519	0	0	0	0	0	0
Streptococcus sp. DP34	0	0	0.0031	0.0101	0.0294	0.1249
Streptococcus sp. JCM 5701	0	0	0	0	0	0
Streptococcus sp. JCM 5702	0	0	0.0017	0.0077	0	0
Streptococcus sp. NJ9704	0	0	0	0	0	0
Streptococcus sp. oral strain T4-E3	0	0	0.0066	0.0295	0	0
Streptococcus sp. oral taxon 064	0.033	0.0797	0.0057	0.018	0.0036	0.0106
Streptococcus sp. oral taxon 070	0	0	0.0058	0.0181	0.0014	0.0043
Streptococcus sp. oral taxon 071	0	0	0.0027	0.0122	0	0
Streptococcus sp. oral taxon 074	0	0	8e-04	0.0038	0	0
Streptococcus sp. oral taxon 431	0.0018	0.0057	0.0061	0.0193	0	0
Streptococcus sp. oral taxon C65	0	0	0	0	0.0019	0.0079
Streptococcus sp. oral taxon E12	0	0	0	0	0	0
Streptococcus sp. Q28-2a	0	0	0	0	0	0
Streptococcus sp. THG-M4	0	0	0.0052	0.0231	0	0
Streptococcus sp. VA10345_05	7e-04	0.0031	0	0	0	0
Streptococcus vestibularis	0	0	0	0	0.0011	0.0045
Tannerella forsythia	0	0	0.0013	0.0057	9e-04	0.0038
Tannerella sp. oral taxon 808	0.0012	0.0051	0	0	6e-04	0.0027
Tannerella sp. oral taxon HOT-286	0.0017	0.0076	0	0	8e-04	0.0034
Tessaracoccus aquimaris	0	0	0	0	0.0041	0.0173
Thermotalea metallivorans	0	0	0	0	4e-04	0.0017
Treponema denticola	0	0	0	0	0.002	0.0085
Treponema maltophilum	0	0	0	0	0	0
Treponema socranskii	0	0	0	0	0.0032	0.0134
Treponema sp. I	0	0	0	0	0	0
Treponema sp. IV	0	0	0	0	0.001	0.0044
Treponema sp. oral taxon 237	0	0	0.0024	0.0109	0.0046	0.0197

Veillonella atypica	0	0	0	0	4e-04	0.0016
Veillonella dispar	0.0078	0.023	0.0021	0.0067	0.0207	0.0559
Veillonella parvula	0.0149	0.0592	0.003	0.0075	0.0057	0.0166
Veillonella ratti	0	0	0	0	0.0023	0.0098
Veillonella sp.	0	0	0	0	4e-04	0.0019
Veillonella sp. ICM51a	0.0013	0.0058	0	0	0	0
Veillonella sp. oral taxon 158	0	0	0	0	0	0
Wandonia haliotis	0	0	0	0	0	0
	**Titanium**					
**Species**	**T0_mean**	**T0_sd**	**T1_mean**	**T1_sd**	**T2_mean**	**T2_sd**
Abiotrophia defectiva	0.0151	0.0327	0	0	0.0084	0.0243
Acetobacteroides hydrogenigenes	0	0	0	0	4e-04	0.0016
Acinetobacter sp. G3DM-29	0	0	0	0	0	0
Actinomyces dentalis	0	0	0.0095	0.0413	4e-04	0.0018
Actinomyces genomosp. C1	0	0	0	0	0	0
Actinomyces gerencseriae	0	0	0	0	0	0
Actinomyces graevenitzii	0	0	0.021	0.0914	0	0
Actinomyces israelii	0	0	0.0666	0.2299	0.118	0.2166
Actinomyces naeslundii	0.0156	0.068	0.0239	0.0907	0.0136	0.0578
Actinomyces oris	0	0	0.0776	0.1838	0.0372	0.1185
Actinomyces provencensis	0	0	0	0	0	0
Actinomyces sp.	0.0108	0.0358	0	0	0	0
Actinomyces sp. 'ARUP UnID 105'	0	0	0	0	0.0075	0.0319
Actinomyces sp. 'ARUP UnID 46'	0	0	0.0049	0.0212	0.0022	0.0063
Actinomyces sp. 'ARUP UnID 51'	0	0	0	0	0.0477	0.2024
Actinomyces sp. 'ARUP UnID 53'	0	0	0.0019	0.0085	0.004	0.0171
Actinomyces sp. 'ARUP UnID 56'	0.0052	0.0225	0.0037	0.0161	0	0
Actinomyces sp. 'ARUP UnID 58'	0.0085	0.0369	0.0083	0.0362	0	0
Actinomyces sp. 'ARUP UnID 59'	0.0044	0.019	0	0	0	0
Actinomyces sp. 'ARUP UnID 62'	0	0	0	0	0	0
Actinomyces sp. 'ARUP UnID 70'	0.005	0.022	0	0	0	0
Actinomyces sp. 'ARUP UnID 71'	0	0	0	0	0	0
Actinomyces sp. 'ARUP UnID 72'	0.0255	0.1112	0	0	0	0
Actinomyces sp. 'ARUP UnID 77'	0	0	0	0	0	0
Actinomyces sp. 'ARUP UnID 89'	0	0	0.017	0.0739	0.004	0.0168
Actinomyces sp. 'ARUP UnID 95'	0	0	0	0	0	0
Actinomyces sp. 11-179	0	0	0	0	0.0221	0.0938
Actinomyces sp. 12-664	0.0141	0.0615	0.0086	0.0375	0.006	0.0254
Actinomyces sp. 13-266	0	0	0	0	0.0026	0.0111
Actinomyces sp. A1	0.0027	0.0117	0	0	0	0
Actinomyces sp. A3	0	0	0.0262	0.1144	0.0103	0.0436
Actinomyces sp. ChDC B197	0.0177	0.0771	0	0	0	0
Actinomyces sp. ChDC B642	0	0	0.0014	0.006	0.0194	0.0657
Actinomyces sp. ChDC B645	0	0	0.0088	0.0382	0.0498	0.2115
Actinomyces sp. ICM39	0	0	0	0	0	0
Actinomyces sp. oral strain Hal-1065	0	0	0	0	0.0013	0.0057

Actinomyces sp. oral taxon 169	0	0	0	0	0.0044	0.0187
Actinomyces sp. oral taxon 170	0	0	0	0	0.0048	0.0203
Actinomyces sp. oral taxon 171	0	0	0.0019	0.0081	0	0
Actinomyces sp. oral taxon 175	0.0176	0.0542	0.0151	0.0484	0.0138	0.0474
Actinomyces sp. oral taxon 525	0	0	0	0	0	0
Actinomyces sp. oral taxon 897	0	0	0.0157	0.0483	0.0187	0.0592
Actinomyces sp. oral taxon A50	0.0055	0.024	0.0175	0.0762	0.0012	0.0049
Actinomyces sp. oral taxon B78	0	0	0	0	0.0082	0.0348
Actinomyces sp. oral taxon E63	0	0	0.0056	0.0243	0	0
Actinomyces sp. R42.11	0	0	0	0	0	0
Actinomyces sp. R42.5	0.0199	0.0615	0	0	0.0105	0.0444
Actinomyces viscosus	0	0	0	0	0	0
Aerococcus christensenii	0	0	0	0	0	0
Aggregatibacter aphrophilus	0.0012	0.0051	0	0	0	0
Aggregatibacter kilianii	0	0	0	0	0	0
Aggregatibacter segnis	0	0	0.0138	0.0414	0.0021	0.0088
Aggregatibacter sp. 316364/07	0.001	0.0045	0	0	0	0
Aggregatibacter sp. HS19_2W_I12	0.0015	0.0048	0	0	0	0
Alishewanella alkalitolerans	0	0	0	0	0	0
Alkaliflexus imshenetskii	0	0	0.0078	0.0271	0	0
Alkalihalobacillus alkalisediminis	0	0	0	0	0	0
Alkalilacustris brevis	0.0143	0.0624	0	0	0	0
Alkalimonas sp. BW86-79	0	0	0	0	0	0
Alkaliphilus hydrothermalis	0	0	0	0	0	0
Alkaliphilus sp.	0	0	0	0	0	0
Alloprevotella Prevotella sp. oral taxon 308	0	0	0	0	0	0
Alloprevotella rava	0	0	0.0012	0.0052	0	0
Alloprevotella tannerae	0.0017	0.0072	0.0015	0.0064	0.0024	0.0101
Anaerobacillus sp.	0	0	0	0	0	0
Anaerobacillus sp. M5-13	0	0	0	0	0	0
Anaerobranca sp. S55_26_2	0	0	0	0	0	0
Anaerocella delicata	0	0	0	0	0	0
Anaerocolumna cellulosilytica	0	0	0	0	0.0015	0.0066
Anaerocolumna xylanovorans	0	0	0.0058	0.0196	0	0
Anaeroglobus geminatus	0	0	0	0	0.0018	0.0043
Anaeromassilibacillus sp. Marseille-P3371	0.0012	0.0053	0	0	0	0
Anaerovorax odorimutans	0	0	0	0	5e-04	0.002
Atopobium deltae	0	0	0	0	0	0
Atopobium parvulum	0	0	0	0	0.0193	0.0763
Atopobium rimae	0	0	0.0253	0.1104	0.0029	0.0085
Atopobium sp. DMCT15023	0	0	0	0	0	0
Atopobium vaginae	0	0	0	0	0.0023	0.0097
Azospirillum sp. AP-500	0	0	5e-04	0.002	3e-04	0.0015
Bacillus cellulosilyticus	0	0	0.0032	0.0098	0	0
Bacillus sp. ANL-isoa2	0	0	0	0	0	0
Bacillus sp. oral taxon C44	0	0	0.0115	0.044	0	0
Bacteroides heparinolyticus	0	0	0	0	0	0
Bacteroides salyersiae	0	0	0	0	0	0

Belliella sp. LW3	0	0	0	0	0	0
Bergeyella sp. AF14	0.0031	0.0136	0.0025	0.0111	0.0052	0.0128
Bergeyella sp. oral taxon 322	0.0064	0.0278	0.0014	0.0061	0.0016	0.0069
Blautia sp. Marseille-P3313	0	0	0.0111	0.0332	0	0
Brevilactibacter sinopodophylli	0.0116	0.0507	0	0	0	0
Bulleidia extructa	0	0	0	0	0	0
Campylobacter concisus	0	0	0	0	0	0
Campylobacter curvus	0.0053	0.0233	0	0	0.0034	0.0091
Campylobacter gracilis	0	0	0.0141	0.0615	0.0101	0.0198
Campylobacter rectus	0	0	0	0	0.0031	0.0115
Campylobacter showae	0.0021	0.0091	0.0013	0.0057	0.0089	0.0271
Campylobacter sp. FOBRC14	0	0	0	0	0	0
Campylobacter sp. oral taxon G43	0	0	0	0	0.0012	0.0051
Candidatus Saccharimonas aalborgensis	0	0	0	0	0	0
Candidatus Saccharimonas sp.	5e-04	0.0021	0.006	0.022	0.0013	0.0046
Capnocytophaga genosp. AHN8471	0	0	0	0	0	0
Capnocytophaga gingivalis	0.0133	0.0431	0.0188	0.065	0.0085	0.0359
Capnocytophaga granulosa	0.0012	0.0053	0	0	0	0
Capnocytophaga leadbetteri	0.0016	0.0071	0	0	0.0051	0.0148
Capnocytophaga ochracea	0.0194	0.0844	0	0	0.0024	0.0057
Capnocytophaga sp.	0.0061	0.0199	0	0	0	0
Capnocytophaga sp. 'ARUP UnID 181'	0	0	0	0	0.002	0.0086
Capnocytophaga sp. 'ARUP UnID 182'	0	0	0	0	0	0
Capnocytophaga sp. 'ARUP UnID 184'	0	0	0	0	5e-04	0.0023
Capnocytophaga sp. AHN10044	0.0035	0.0153	0.0055	0.0241	0.002	0.0085
Capnocytophaga sp. AHN9756	0	0	0	0	0	0
Capnocytophaga sp. FDAARGOS_737	0.0087	0.0256	0	0	0.0029	0.0118
Capnocytophaga sp. FVAMC 7623	0	0	0	0	0.0038	0.0163
Capnocytophaga sp. oral taxon 336	0	0	0	0	0.0045	0.0132
Capnocytophaga sp. oral taxon 338	0	0	0.0012	0.0051	5e-04	0.0023
Capnocytophaga sp. oral taxon 864	0	0	0	0	0.0012	0.005
Capnocytophaga sp. oral taxon A48	0.0017	0.0074	0	0	0	0
Capnocytophaga sp. oral taxon B29	0	0	0	0	0	0
Capnocytophaga sputigena	0.052	0.1495	0	0	0.0037	0.0117
Cardiobacterium sp. 'ARUP UnID 187'	0	0	0	0	0	0
Catonella morbi	0	0	0	0	9e-04	0.004
Catonella sp. oral taxon F17	0	0	0	0	0.001	0.0031
Cellulomonas hominis	0.0031	0.0135	0.0019	0.0084	0	0
Centipeda sp. oral taxon F73	0	0	0	0	0	0
Christensenella minuta	0	0	0	0	0	0
Clostridium sp. 6-44	0	0	7e-04	0.003	0	0
Corynebacterium durum	0	0	0	0	0	0
Corynebacterium matruchotii	0.0017	0.0074	0	0	0	0
Corynebacterium sp. oral taxon B00	0	0	0	0	0	0
Corynebacterium sp. ZT10-3	0	0	0	0	0	0
Cryptobacterium curtum	0	0	0	0	0	0
Desulfobulbus oralis	0	0	0	0	0	0
Desulfonispora thiosulfatigenes	0	0	0	0	2e-04	0.001
Desulfovibrio sp. feline oral taxon 347	0	0	0	0	4e-04	0.0015

Dialister invisus	0	0	0	0	0.0015	0.0036
Dialister micraerophilus	0	0	0	0	5e-04	0.0022
Dialister pneumosintes	0	0	0	0	8e-04	0.0031
Dialister sp. E2_20	0	0	0	0	5e-04	0.0022
Dialister sp. oral taxon C66	0	0	0.0015	0.0065	4e-04	0.0016
Dorea Candidatus Dorea massiliensis	0	0	0.019	0.0481	0.0123	0.0407
Dysgonomonas sp. Marseille-P4677	0	0	0.0033	0.0146	0.0045	0.0192
Eggerthia catenaformis	0	0	0	0	0	0
Eikenella corrodens	0	0	0	0	0	0
Eikenella longinqua	0	0	0	0	0	0
Enterococcus italicus	0	0	0	0	0	0
Enterococcus sp. oral taxon A43	0	0	0	0	0	0
Erythrobacter sp. MED13	0	0	0.0104	0.0455	0	0
Escherichia coli	0	0	0	0	0	0
Eubacterium sp.	0	0	0	0	8e-04	0.0023
Eubacterium sp. oral strain A35MT	0	0	0	0	3e-04	0.0011
Eubacterium sp. oral taxon G32	0	0	0	0	0	0
Faecalicatena fissicatena	6e-04	0.0024	0.0047	0.0206	0	0
Filifactor alocis	0	0	0	0	0.0018	0.0075
Flavobacterium sp.	0	0	0	0	0	0
Flexilinea flocculi	0	0	0	0	0.0055	0.0234
Fretibacterium fastidiosum	0	0	0.0078	0.0338	0.0019	0.0061
Fretibacterium sp. feline oral taxon 223	0	0	0	0	0.005	0.0162
Fusibacter paucivorans	0	0	0	0	8e-04	0.0023
Fusobacterium hwasookii	0.0013	0.004	0.003	0.0131	6e-04	0.0025
Fusobacterium naviforme	0	0	0	0	2e-04	0.001
Fusobacterium nucleatum	0.005	0.01	0.0062	0.0186	0.0169	0.0278
Fusobacterium pseudoperiodonticum	0.0022	0.0052	0	0	9e-04	0.0031
Fusobacterium sp. oral taxon 203	0	0	0	0	9e-04	0.0037
Gemella haemolysans	0.0063	0.0257	0	0	0.0055	0.0235
Gemella morbillorum	0	0	0	0	4e-04	0.0013
Gemmobacter sp. YIM 102744-1	0	0	0.0067	0.0293	0.0012	0.0038
Geosporobacter ferrireducens	0	0	0	0	0	0
Granulicatella adiacens	0	0	0.0171	0.052	0.036	0.0846
Granulicatella elegans	0	0	0	0	0.0039	0.0165
Haematobacter massiliensis	0	0	0	0	0	0
Haemophilus haemolyticus	0	0	4e-04	0.002	7e-04	0.0031
Haemophilus influenzae	0	0	0	0	0	0
Haemophilus parahaemolyticus	0	0	0	0	0	0
Haemophilus parainfluenzae	0.0528	0.1508	0.0091	0.0324	0.0146	0.0493
Haemophilus paraphrohaemolyticus	0.0018	0.008	0	0	0	0
Haemophilus sp. CCUG 13929	0	0	0	0	0.0028	0.0117
Haemophilus sp. CCUG 23622	0	0	0	0	0	0
Haemophilus sp. oral taxon 036	0	0	0	0	0	0
Haemophilus sputorum	0	0	0	0	2e-04	8e-04
Ihubacter massiliensis	0	0	0	0	0	0
Johnsonella ignava	0	0	0	0	0	0
Kingella denitrificans	0.0319	0.0876	0	0	0	0
Kingella oralis	0.036	0.1569	0	0	0	0

Lachnoanaerobaculum cf. saburreum oral strain C27KA	0	0	0.0158	0.0518	0.0021	0.0089
Lachnoanaerobaculum gingivalis	0.0059	0.0256	0.0016	0.0071	0	0
Lachnoanaerobaculum orale	0	0	0.0081	0.0354	0	0
Lachnoanaerobaculum saburreum	0	0	0	0	0	0
Lacrimispora saccharolytica	0	0	0.0012	0.005	3e-04	0.0011
Lactobacillus delbrueckii	0.0075	0.0329	0	0	0	0
Lactobacillus fermentum	0	0	0	0	0.0031	0.0133
Lactobacillus gasseri	0	0	0	0	0	2e-04
Lactobacillus kitasatonis	0	0	0.0011	0.0049	7e-04	0.0031
Lactobacillus paracasei	0	0	0	0	0.001	0.0041
Lactobacillus reuteri	0	0	0	0	0	0
Lactobacillus rhamnosus	0	0	0	0	7e-04	0.0029
Lactobacillus salivarius	0.0044	0.0136	6e-04	0.0024	0	0
Lactobacillus sp. 46-211	0	0	0	0	0.0012	0.0051
Lactobacillus vaginalis	0	0	0	0	3e-04	0.0014
Lautropia mirabilis	0.0541	0.1949	0.0205	0.0894	0	0
Lawsonella clevelandensis	0	0	0	0	0	0
Leptotrichia buccalis	0.0076	0.0331	0	0	0	0
Leptotrichia genomosp. C1	2e-04	0.001	0	0	0.0012	0.0052
Leptotrichia massiliensis	0	0	0	0	0	0
Leptotrichia shahii	0	0	0	0	0	0
Leptotrichia sp.	0.0014	0.006	0	0	0	0
Leptotrichia sp. oral taxon 212	0.0015	0.0046	0	0	1e-04	3e-04
Leptotrichia sp. oral taxon 215	7e-04	0.003	0	0	0.0015	0.0043
Leptotrichia sp. oral taxon 217	0.0035	0.0108	4e-04	0.0018	0.0046	0.0173
Leptotrichia sp. oral taxon 218	0	0	0	0	0	0
Leptotrichia sp. oral taxon 219	2e-04	7e-04	0	0	0.001	0.0041
Leptotrichia sp. oral taxon 221	0	0	0	0	0	0
Leptotrichia sp. oral taxon 225	0.0044	0.0095	0	0	0.0013	0.0054
Leptotrichia sp. oral taxon 392	0	0	0	0	6e-04	0.0024
Leptotrichia sp. oral taxon 463	4e-04	0.002	8e-04	0.0034	0	0
Leptotrichia sp. oral taxon 498	0	0	0	0	0	0
Leptotrichia trevisanii	0.0019	0.0048	0	0	0	0
Leptotrichia wadei	6e-04	0.0019	0.0027	0.0094	0.0084	0.0275
Mageeibacillus indolicus	0	0	0	0	0	0
Megasphaera genomosp. C1	0	0	0	0	0	0
Megasphaera micronuciformis	0.0034	0.0148	0	0	0	0
Methylobacterium haplocladii	0	0	0	0	0	0
Microbacterium sp. NDK-63	7e-04	0.003	0	0	0	0
Microcella alkaliphila	0	0	0	0	0	0
Mogibacterium timidum	0	0	0	0	0	0
Mongoliitalea sp.	0	0	0.007	0.0305	0	0
Moryella sp. KHD1	0.0042	0.0182	0.0146	0.0526	8e-04	0.0033
Mycoplasma faucium	0	0	0	0	0	0
Mycoplasma hyorhinis	0	0	0	0	0.0016	0.0069
Mycoplasma salivarium	0	0	0	0	0	0
Natronohydrobacter thiooxidans	0.0062	0.0269	0	0	0	0
Neisseria bacilliformis	0	0	0.0031	0.0136	0	0
Neisseria elongata	0	0	0	0	0.0012	0.0052

Neisseria flava	0	0	0	0	0	0
Neisseria flavescens	0	0	0	0	0	0
Neisseria sp. 'ARUP UnID 432'	0	0	0	0	0	0
Neisseria sp. oral taxon 014	0	0	0	0	0.0016	0.0066
Neisseria sp. oral taxon 499	0	0	0	0	0	0
Neisseria subflava	9e-04	0.0039	0	0	0.0044	0.0187
Nesterenkonia sp.	0	0	0	0	0.0039	0.0164
Nibricoccus aquaticus	0	0	0	0	0	0
Nitrincola sp. E-044	0	0	0	0	0	0
Okadaella gastrococcus	0	0	0.0062	0.0272	6e-04	0.0026
Olsenella sp. F0004	0	0	0	0	0	0
Oribacterium sp. oral taxon 078	0	0	0.001	0.0044	0	0
Paludibacter propionicigenes	0.0029	0.0086	0	0	0.0091	0.0291
Parabacteroides sp. Marseille-P3160	0	0	0.0196	0.0649	0.0012	0.005
Paracoccus sp.	0	0	0.0018	0.0079	0	0
Pararhodobacter sp.	0	0	0.0062	0.0155	0	0
Parvimonas micra	4e-04	0.0019	6e-04	0.0027	0.0011	0.0031
Parvimonas sp. canine oral taxon 102	0	0	0	0	0	0
Peptoanaerobacter [Eubacterium] yurii	0	0	0	0	0	0
Peptococcus sp. oral taxon 168	0	0	0	0	0	0
Peptoniphilus sp. oral taxon 836	0	0	0	0	0	0
Peptostreptococcus sp. CCUG 42997	0	0	0	0	0.0037	0.0156
Peptostreptococcus sp. oral taxon 113	0	0	0	0	0	0
Peptostreptococcus stomatis	0	0	0	0	0.0028	0.0121
Phocaeicola abscessus	0	0	0	0	0	0
Porphyromonas catoniae	9e-04	0.004	0	0	0.0021	0.0089
Porphyromonas endodontalis	0	0	0	0	0.0076	0.018
Porphyromonas gingivalis	0	0	0	0	0.0146	0.04
Porphyromonas pasteri	0.0117	0.0325	0.0064	0.0279	0.0013	0.0057
Porphyromonas sp. oral taxon 275	0.0016	0.0069	0	0	0	0
Porphyromonas sp. oral taxon 278	0	0	0	0	0	0
Prevotella baroniae	0	0	0	0	0.0011	0.0045
Prevotella conceptionensis	0	0	0	0	0	0
Prevotella dentalis	0	0	0	0	0	0
Prevotella denticola	0	0	0	0	0.0015	0.0065
Prevotella genomosp. C1	0	0	0.0021	0.0093	0	0
Prevotella genomosp. P6	0	0	0	0	0	0
Prevotella intermedia	0.0123	0.0538	0	0	8e-04	0.0033
Prevotella koreensis	0	0	0	0	0	0
Prevotella loescheii	0.002	0.0059	0.011	0.048	0	0
Prevotella maculosa	0	0	0.0016	0.0072	0	0
Prevotella melaninogenica	0.033	0.0674	0.0627	0.2159	0.022	0.0599
Prevotella micans	0	0	0	0	0	0
Prevotella multiformis	0	0	0	0	0	0
Prevotella nanceiensis	0	0	0	0	0	0
Prevotella oralis	0	0	0	0	0.0028	0.0117
Prevotella oris	0.0027	0.0082	0.0056	0.0244	0.0153	0.0278
Prevotella oulorum	0	0	0.0037	0.016	0.0018	0.0078
Prevotella pallens	0	0	0	0	0	0
Prevotella salivae	0.0655	0.2081	0.0189	0.0424	0.0052	0.0114

Prevotella sp. 8404125	9e-04	0.004	0	0	2e-04	0.001
Prevotella sp. CM38	0	0	0	0	4e-04	0.0017
Prevotella sp. oral taxon 292	0	0	0	0	8e-04	0.0034
Prevotella sp. oral taxon 300	0	0	0.0012	0.0053	0.0023	0.0071
Prevotella sp. oral taxon 303	0	0	0	0	0	0
Prevotella sp. oral taxon 314	0	0	0.0033	0.0143	0.0029	0.0085
Prevotella sp. oral taxon 317	0	0	0.0023	0.0098	3e-04	0.0012
Prevotella sp. oral taxon 472	0.0023	0.0099	0	0	0	0
Prevotella sp. oral taxon 475	9e-04	0.004	5e-04	0.0022	0	0
Prevotella sp. oral taxon 515	0	0	0	0	0	0
Prevotella sp. oral taxon G60	0	0	0	0	0	0
Prosthecomicrobium sp. 'ARUP UnID 533'	0	0	0.0043	0.0186	0	0
Pseudoleptotrichia goodfellowii	0	0	0	0	0.0046	0.0196
Pseudomonas tarimensis	0	0	0	0	0	0
Pseudomonas xinjiangensis	0	0	0.0018	0.008	0.0054	0.0231
Pseudopropionibacterium massiliense	0	0	0	0	0	0
Pseudopropionibacterium propionicum	0.0073	0.0319	0.0041	0.0126	0	0
Pseudoramibacter alactolyticus	0	0	0	0	0.0083	0.0351
Pseudoruminococcus massiliensis	0	0	0.0041	0.018	0	0
Rhodobacter sp. CCP-1	0	0	0	0	0	0
Roseomonas sp.	0	0	0.0098	0.0253	0	0
Rothia dentocariosa	0	0	0	0	0	0
Rothia mucilaginosa	0	0	0	0	0	0
Schaalia cardiffensis	0	0	0	0	0.0019	0.008
Schaalia georgiae	0	0	0	0	0	0
Schaalia meyeri	0.0033	0.0144	0	0	0.0021	0.0088
Schaalia odontolytica	0.0323	0.0869	0	0	0.0221	0.0559
Schwartzia sp. canine oral taxon 042	0	0	0	0	0.0026	0.0096
Selenomonas artemidis	0	0	7e-04	0.0029	0.0024	0.0101
Selenomonas dianae	0	0	0	0	4e-04	0.0017
Selenomonas infelix	0	0	0	0	3e-04	0.0013
Selenomonas noxia	2e-04	8e-04	9e-04	0.0032	0.0043	0.0108
Selenomonas sp. oral taxon 126	0	0	0	0	5e-04	0.0021
Selenomonas sp. oral taxon 134	0	0	8e-04	0.0034	7e-04	0.0028
Selenomonas sp. oral taxon 137	0	0	0	0	0	0
Selenomonas sp. oral taxon 138	0	0	0	0	0	0
Selenomonas sp. oral taxon 149	0	0	4e-04	0.0018	0	0
Selenomonas sp. oral taxon 920	0	0	0	0	2e-04	9e-04
Selenomonas sp. oral taxon F19	0	0	0	0	2e-04	0.001
Selenomonas sp. oral taxon F20	0	0	3e-04	0.0015	0	0
Selenomonas sp. oral taxon F21	0	0	0	0	0	0
Selenomonas sp. oral taxon F72	0	0	0	0	4e-04	0.0012
Selenomonas sp. oral taxon F81	0	0	0	0	4e-04	0.0012
Selenomonas sp. oral taxon F82	0	0	7e-04	0.0025	0.0012	0.0031
Selenomonas sp. oral taxon G00	0	0	0	0	5e-04	0.002
Selenomonas sp. oral taxon G67	0.0039	0.017	0	0	0	0
Selenomonas sp. oral taxon H64	0	0	6e-04	0.0022	5e-04	0.002
Selenomonas sp. oral taxon H65	0	0	0	0	0	0
Selenomonas sp. oral taxon H66	0	0	3e-04	0.001	0	0

Selenomonas sputigena	0.0056	0.0245	0.0018	0.0077	0.0017	0.0057
Shuttleworthia satelles	0	0	0	0	0	0
Slackia exigua	0	0	0	0	0.0031	0.0091
Solobacterium moorei	0	0	0	0	0.0012	0.0043
Staphylococcus aureus	0	0	0	0	0	0
Streptococcus anginosus	0.0123	0.0537	0.0223	0.0781	0.006	0.0205
Streptococcus australis	0.003	0.0075	0.0019	0.0084	0.0051	0.0151
Streptococcus constellatus	0	0	0	0	9e-04	0.0038
Streptococcus cristatus	0	0	0.0021	0.009	0	0
Streptococcus downei	0.0107	0.038	0.0027	0.0086	0	0
Streptococcus gordonii	0.0055	0.0241	0.0112	0.0488	0.0053	0.0115
Streptococcus gwangjuense	0	0	0.0139	0.0462	0	0
Streptococcus infantis	0	0	0.0203	0.0886	0	0
Streptococcus intermedius	0.0335	0.1126	0.002	0.0088	0.0056	0.0215
Streptococcus mitis	0.0414	0.1043	0.0238	0.0601	0.0185	0.0619
Streptococcus mutans	0	0	0.004	0.0174	0.0019	0.0082
Streptococcus oralis	0.0269	0.1159	9e-04	0.0037	0.003	0.0112
Streptococcus parasanguinis	0.006	0.017	0.0059	0.0164	0.012	0.0337
Streptococcus periodonticum	0	0	0	0	0	0
Streptococcus peroris	0	0	0	0	0	0
Streptococcus pneumoniae	6e-04	0.0026	0	0	0	0
Streptococcus salivarius	0	0	0	0	0.0024	0.0102
Streptococcus sanguinis	0.0551	0.143	0	0	0.0111	0.0269
Streptococcus sp.	0.0035	0.0153	0.002	0.0089	5e-04	0.0021
Streptococcus sp. 'ARUP UnID 614'	0	0	0	0	0	0
Streptococcus sp. 'ARUP UnID 639'	0	0	0.0048	0.0208	0	0
Streptococcus sp. 10aMclG2	0	0	0	0	0	0
Streptococcus sp. 10aVMg2	0	0	0	0	0.0018	0.0077
Streptococcus sp. 13aVMg2	0	0	0.0044	0.0157	0.0021	0.0088
Streptococcus sp. A12	0	0	0.0033	0.0099	0.0027	0.0088
Streptococcus sp. AS14	0	0	0	0	0.0073	0.0309
Streptococcus sp. B2	0.0021	0.009	0	0	0	0
Streptococcus sp. ChDC B366	0.0019	0.0081	7e-04	0.0029	0	0
Streptococcus sp. ChDC B519	0	0	0	0	0.0044	0.0189
Streptococcus sp. DP34	0.014	0.0612	0	0	7e-04	0.0031
Streptococcus sp. JCM 5701	0	0	0	0	7e-04	0.0028
Streptococcus sp. JCM 5702	0	0	0	0	2e-04	7e-04
Streptococcus sp. NJ9704	5e-04	0.0021	0	0	0	0
Streptococcus sp. oral strain T4-E3	0	0	0	0	0.0012	0.0051
Streptococcus sp. oral taxon 064	0.0017	0.0051	0.0342	0.1238	0.0145	0.0584
Streptococcus sp. oral taxon 070	0.0035	0.0153	0.0037	0.0154	0.0011	0.0042
Streptococcus sp. oral taxon 071	0	0	0	0	0	0
Streptococcus sp. oral taxon 074	0	0	0	0	0	0
Streptococcus sp. oral taxon 431	9e-04	0.004	0	0	5e-04	0.002
Streptococcus sp. oral taxon C65	0	0	0	0	0.0014	0.0058
Streptococcus sp. oral taxon E12	0.0053	0.0164	0	0	0	0
Streptococcus sp. Q28-2a	0.001	0.0045	0	0	0	0
Streptococcus sp. THG-M4	0	0	0	0	0	0
Streptococcus sp. VA10345_05	5e-04	0.0023	0	0	0	0
Streptococcus vestibularis	0	0	0	0	0.0014	0.0061

Tannerella forsythia	0	0	0	0	0.0014	0.006
Tannerella sp. oral taxon 808	0	0	0	0	0	0
Tannerella sp. oral taxon HOT-286	0	0	0	0	0.0011	0.0045
Tessaracoccus aquimaris	0	0	0	0	0	0
Thermotalea metallivorans	0	0	0	0	0	0
Treponema denticola	0	0	0.0023	0.0099	0.0022	0.0091
Treponema maltophilum	0	0	0	0	0	0
Treponema socranskii	0	0	0	0	0.0051	0.0122
Treponema sp. I	0	0	0	0	7e-04	0.003
Treponema sp. IV	0	0	0	0	6e-04	0.0026
Treponema sp. oral taxon 237	0	0	0	0	0.0013	0.0057
Veillonella atypica	0	0	0	0	7e-04	0.003
Veillonella dispar	8e-04	0.0025	0	0	0.0104	0.0245
Veillonella parvula	0.002	0.0069	0.0051	0.0145	0.0087	0.0171
Veillonella ratti	0	0	0	0	8e-04	0.0034
Veillonella sp.	0	0	0	0	0	0
Veillonella sp. ICM51a	0	0	0.0035	0.0153	0.001	0.004
Veillonella sp. oral taxon 158	0	0	0	0	0	0
Wandonia haliotis	0	0	0.003	0.0131	0	0

	Tooth					
**Species**	**T0_mean**	**T0_sd**	**T1_mean**	**T1_sd**	**T2_mean**	**T2_sd**
Abiotrophia defectiva	0	0	0	0	0	0
Acetobacteroides hydrogenigenes	0	0	0	0	0	0
Acinetobacter sp. G3DM-29	0.008	0.0195	0	0	0	0
Actinomyces dentalis	0	0	0	0	0	0
Actinomyces genomosp. C1	0	0	0	0	0.0286	0.0701
Actinomyces gerencseriae	0.0149	0.0366	0	0	0	0
Actinomyces graevenitzii	0	0	0	0	0	0
Actinomyces israelii	0	0	0	0	0	0
Actinomyces naeslundii	0	0	0	0	0	0
Actinomyces oris	0	0	0.0388	0.0699	0	0
Actinomyces provencensis	0	0	0	0	0.0359	0.0879
Actinomyces sp.	0	0	0	0	0.0206	0.0506
Actinomyces sp. 'ARUP UnID 105'	0	0	0	0	0	0
Actinomyces sp. 'ARUP UnID 46'	0	0	0	0	0	0
Actinomyces sp. 'ARUP UnID 51'	0	0	0	0	0	0
Actinomyces sp. 'ARUP UnID 53'	0	0	0	0	0	0
Actinomyces sp. 'ARUP UnID 56'	0	0	0	0	0	0
Actinomyces sp. 'ARUP UnID 58'	0	0	0	0	0	0
Actinomyces sp. 'ARUP UnID 59'	0	0	0	0	0	0
Actinomyces sp. 'ARUP UnID 62'	0	0	0	0	0	0
Actinomyces sp. 'ARUP UnID 70'	0	0	0	0	0	0
Actinomyces sp. 'ARUP UnID 71'	0.0241	0.0591	0	0	0	0
Actinomyces sp. 'ARUP UnID 72'	0	0	0	0	0	0
Actinomyces sp. 'ARUP UnID 77'	0.0321	0.0787	0	0	0	0
Actinomyces sp. 'ARUP UnID 89'	0	0	0.033	0.0808	0.0048	0.0117

Actinomyces sp. 'ARUP UnID 95'	0	0	0	0	0	0
Actinomyces sp. 11-179	0	0	0	0	0	0
Actinomyces sp. 12-664	0	0	0.013	0.0317	0	0
Actinomyces sp. 13-266	0.032	0.0784	0	0	0	0
Actinomyces sp. A1	0	0	0	0	0	0
Actinomyces sp. A3	0	0	0	0	0	0
Actinomyces sp. ChDC B197	0.0898	0.22	0	0	0	0
Actinomyces sp. ChDC B642	0	0	0	0	0	0
Actinomyces sp. ChDC B645	0	0	0	0	0	0
Actinomyces sp. ICM39	0	0	0	0	0	0
Actinomyces sp. oral strain Hal-1065	0	0	0	0	0	0
Actinomyces sp. oral taxon 169	0	0	0	0	0	0
Actinomyces sp. oral taxon 170	0	0	0	0	0	0
Actinomyces sp. oral taxon 171	0	0	0	0	0	0
Actinomyces sp. oral taxon 175	0	0	0	0	0	0
Actinomyces sp. oral taxon 525	0	0	0	0	0	0
Actinomyces sp. oral taxon 897	0	0	0	0	0	0
Actinomyces sp. oral taxon A50	0	0	0	0	0	0
Actinomyces sp. oral taxon B78	0	0	0	0	0	0
Actinomyces sp. oral taxon E63	0	0	0	0	0	0
Actinomyces sp. R42.11	0	0	0	0	0	0
Actinomyces sp. R42.5	0	0	0	0	0	0
Actinomyces viscosus	0	0	0	0	0	0
Aerococcus christensenii	0	0	0	0	0	0
Aggregatibacter aphrophilus	0	0	0	0	0	0
Aggregatibacter kilianii	0	0	0	0	0	0
Aggregatibacter segnis	0	0	0	0	0	0
Aggregatibacter sp. 316364/07	0	0	0	0	0	0
Aggregatibacter sp. HS19_2W_I12	0	0	0	0	0	0
Alishewanella alkalitolerans	0	0	0	0	0	0
Alkaliflexus imshenetskii	0	0	0	0	0.0075	0.0117
Alkalihalobacillus alkalisediminis	0	0	0	0	0	0
Alkalilacustris brevis	0.0081	0.02	0	0	0	0
Alkalimonas sp. BW86-79	0	0	0	0	0	0
Alkaliphilus hydrothermalis	0	0	0	0	0	0
Alkaliphilus sp.	0	0	0	0	0	0
Alloprevotella Prevotella sp. oral taxon 308	0	0	0	0	0	0
Alloprevotella rava	0	0	0	0	0	0
Alloprevotella tannerae	0.0057	0.0139	0.0112	0.0274	0.0023	0.0056
Anaerobacillus sp.	0	0	0	0	0	0
Anaerobacillus sp. M5-13	0	0	0	0	0	0
Anaerobranca sp. S55_26_2	0	0	0	0	0.0022	0.0054
Anaerocella delicata	0	0	0.0117	0.0287	0.0067	0.0163
Anaerocolumna cellulosilytica	0	0	0	0	0	0
Anaerocolumna xylanovorans	0	0	0	0	0	0
Anaeroglobus geminatus	0	0	0	0	0.0057	0.0098
Anaeromassilibacillus sp. Marseille-P3371	0	0	0	0	0	0
Anaerovorax odorimutans	0	0	0	0	0	0

Atopobium deltae	0	0	0	0	0	0
Atopobium parvulum	0	0	0	0	0	0
Atopobium rimae	0	0	0	0	0.0038	0.0092
Atopobium sp. DMCT15023	0	0	0	0	0.007	0.0172
Atopobium vaginae	0	0	0	0	0	0
Azospirillum sp. AP-500	0	0	0	0	0	0
Bacillus cellulosilyticus	0	0	0	0	0.0037	0.006
Bacillus sp. ANL-isoa2	0	0	0	0	0.0159	0.0364
Bacillus sp. oral taxon C44	0	0	0	0	0.008	0.011
Bacteroides heparinolyticus	0	0	0	0	0	0
Bacteroides salyersiae	0	0	0.0018	0.0045	0.0018	0.0044
Belliella sp. LW3	0	0	0	0	0.0087	0.0214
Bergeyella sp. AF14	0	0	0	0	0	0
Bergeyella sp. oral taxon 322	0	0	0	0	0	0
Blautia sp. Marseille-P3313	0	0	0	0	0.0103	0.016
Brevilactibacter sinopodophylli	0	0	0	0	0	0
Bulleidia extructa	0.001	0.0025	0	0	0	0
Campylobacter concisus	0	0	0	0	0	0
Campylobacter curvus	0	0	0.0558	0.1223	0	0
Campylobacter gracilis	0	0	0	0	0	0
Campylobacter rectus	0.0232	0.0426	0	0	0.014	0.0343
Campylobacter showae	0	0	0	0	0	0
Campylobacter sp. FOBRC14	0	0	0	0	0	0
Campylobacter sp. oral taxon G43	0	0	0	0	0	0
Candidatus Saccharimonas aalborgensis	0	0	0.0068	0.0116	0.0088	0.0215
Candidatus Saccharimonas sp.	0.0058	0.0141	0.0091	0.0143	0.0062	0.0151
Capnocytophaga genosp. AHN8471	0	0	0.0089	0.0219	0	0
Capnocytophaga gingivalis	0.0068	0.0168	0	0	0	0
Capnocytophaga granulosa	0	0	0.0041	0.01	0	0
Capnocytophaga leadbetteri	0	0	0.0093	0.0228	0	0
Capnocytophaga ochracea	0	0	0.002	0.0049	0	0
Capnocytophaga sp.	0	0	0	0	0	0
Capnocytophaga sp. 'ARUP UnID 181'	0	0	0	0	0	0
Capnocytophaga sp. 'ARUP UnID 182'	0	0	0	0	0	0
Capnocytophaga sp. 'ARUP UnID 184'	0	0	0	0	0	0
Capnocytophaga sp. AHN10044	0	0	0	0	0	0
Capnocytophaga sp. AHN9756	0	0	0	0	0	0
Capnocytophaga sp. FDAARGOS_737	0	0	0	0	0	0
Capnocytophaga sp. FVAMC 7623	0	0	0.0047	0.0116	0	0
Capnocytophaga sp. oral taxon 336	0	0	0	0	0	0
Capnocytophaga sp. oral taxon 338	0	0	0	0	0	0
Capnocytophaga sp. oral taxon 864	0	0	0	0	0	0
Capnocytophaga sp. oral taxon A48	0	0	0	0	0	0
Capnocytophaga sp. oral taxon B29	0	0	0	0	0	0
Capnocytophaga sputigena	0.0071	0.0173	0.0135	0.033	0	0
Cardiobacterium sp. 'ARUP UnID 187'	0	0	0	0	0	0
Catonella morbi	0	0	0	0	0	0
Catonella sp. oral taxon F17	0	0	0	0	0	0
Cellulomonas hominis	0	0	0	0	0	0

Centipeda sp. oral taxon F73	0	0	0	0	0	0
Christensenella minuta	0.0034	0.0083	0.002	0.0049	0	0
Clostridium sp. 6-44	0	0	0	0	0	0
Corynebacterium durum	0.0134	0.0327	0	0	0	0
Corynebacterium matruchotii	0	0	0.0051	0.0126	0	0
Corynebacterium sp. oral taxon B00	0	0	0.0128	0.0315	0	0
Corynebacterium sp. ZT10-3	0	0	0	0	0.0026	0.0064
Cryptobacterium curtum	0	0	0	0	0	0
Desulfobulbus oralis	0	0	0.0059	0.0144	0	0
Desulfonispora thiosulfatigenes	0	0	0	0	0.0034	0.0053
Desulfovibrio sp. feline oral taxon 347	0	0	0	0	0	0
Dialister invisus	0	0	0	0	4e-04	0.001
Dialister micraerophilus	0	0	0	0	0	0
Dialister pneumosintes	0	0	0	0	0	0
Dialister sp. E2_20	0	0	0	0	0	0
Dialister sp. oral taxon C66	0	0	0	0	0	0
Dorea Candidatus Dorea massiliensis	0	0	0	0	0.0937	0.1262
Dysgonomonas sp. Marseille-P4677	0	0	0	0	0.0106	0.026
Eggerthia catenaformis	0	0	0	0	0	0
Eikenella corrodens	0.0178	0.0436	0	0	0	0
Eikenella longinqua	0	0	0	0	0	0
Enterococcus italicus	0	0	0	0	0.003	0.0073
Enterococcus sp. oral taxon A43	0	0	0	0	0.0062	0.0096
Erythrobacter sp. MED13	0	0	0	0	0	0
Escherichia coli	0	0	0	0	0	0
Eubacterium sp.	0.001	0.0025	0	0	0.0055	0.0134
Eubacterium sp. oral strain A35MT	0	0	0	0	0.0034	0.0077
Eubacterium sp. oral taxon G32	0.0051	0.0125	0.0046	0.0113	0	0
Faecalicatena fissicatena	0	0	0	0	0.0074	0.0126
Filifactor alocis	0	0	0	0	0	0
Flavobacterium sp.	0	0	0	0	0.0036	0.0087
Flexilinea flocculi	0.0037	0.0091	0	0	0	0
Fretibacterium fastidiosum	0.0098	0.0156	0.0046	0.0113	0.0015	0.0036
Fretibacterium sp. feline oral taxon 223	0.0152	0.0354	0.0034	0.0082	9e-04	0.0021
Fusibacter paucivorans	0.0043	0.0067	0.0061	0.0103	0	0
Fusobacterium hwasookii	0	0	0	0	0	0
Fusobacterium naviforme	0	0	0	0	0	0
Fusobacterium nucleatum	0.0457	0.0727	0.0908	0.1077	0.029	0.0411
Fusobacterium pseudoperiodonticum	0	0	0	0	0	0
Fusobacterium sp. oral taxon 203	0	0	0.0053	0.0129	0.0062	0.0151
Gemella haemolysans	0.0034	0.0084	0	0	0	0
Gemella morbillorum	0	0	0	0	0	0
Gemmobacter sp. YIM 102744-1	0	0	0	0	0.0065	0.016
Geosporobacter ferrireducens	0	0	0	0	0	0
Granulicatella adiacens	0	0	0	0	0	0
Granulicatella elegans	0	0	0	0	0	0
Haematobacter massiliensis	0.0039	0.0095	0	0	0	0
Haemophilus haemolyticus	0	0	0	0	0	0
Haemophilus influenzae	0	0	0	0	0	0
Haemophilus parahaemolyticus	0	0	0	0	0.0022	0.0054

Haemophilus parainfluenzae	0.0321	0.0454	0	0	0.0037	0.009
Haemophilus paraphrohaemolyticus	0	0	0	0	0	0
Haemophilus sp. CCUG 13929	0	0	0	0	0	0
Haemophilus sp. CCUG 23622	0	0	0	0	0	0
Haemophilus sp. oral taxon 036	0	0	0	0	0	0
Haemophilus sputorum	0	0	0	0	0	0
Ihubacter massiliensis	0	0	0	0	0	0
Johnsonella ignava	0	0	0	0	0	0
Kingella denitrificans	0	0	0	0	0	0
Kingella oralis	0	0	0	0	0	0
Lachnoanaerobaculum cf. saburreum oral strain C27KA	0	0	0	0	0	0
Lachnoanaerobaculum gingivalis	0	0	0	0	0	0
Lachnoanaerobaculum orale	0	0	0	0	0	0
Lachnoanaerobaculum saburreum	0	0	0	0	0	0
Lacrimispora saccharolytica	0	0	0	0	0	0
Lactobacillus delbrueckii	0	0	0	0	0	0
Lactobacillus fermentum	0	0	0	0	0	0
Lactobacillus gasseri	0	0	0	0	0	0
Lactobacillus kitasatonis	0.0121	0.0297	0	0	0	0
Lactobacillus paracasei	0	0	0	0	0	0
Lactobacillus reuteri	0	0	0	0	0	0
Lactobacillus rhamnosus	0	0	0	0	0	0
Lactobacillus salivarius	0	0	0	0	0	0
Lactobacillus sp. 46-211	0	0	0	0	0	0
Lactobacillus vaginalis	0	0	0	0	0	0
Lautropia mirabilis	0.0137	0.0335	0	0	0.0137	0.0336
Lawsonella clevelandensis	0	0	0	0	9e-04	0.0022
Leptotrichia buccalis	0.0121	0.0298	0.0016	0.004	0	0
Leptotrichia genomosp. C1	0.0048	0.0119	0	0	0	0
Leptotrichia massiliensis	0	0	0	0	0	0
Leptotrichia shahii	0	0	0.0133	0.0325	0	0
Leptotrichia sp.	0	0	0.0046	0.0113	0	0
Leptotrichia sp. oral taxon 212	0	0	0	0	0	0
Leptotrichia sp. oral taxon 215	0	0	0	0	0	0
Leptotrichia sp. oral taxon 217	0	0	0	0	0	0
Leptotrichia sp. oral taxon 218	0	0	0	0	0	0
Leptotrichia sp. oral taxon 219	0	0	0	0	0	0
Leptotrichia sp. oral taxon 221	0	0	0	0	0	0
Leptotrichia sp. oral taxon 225	0.0038	0.0092	0	0	0	0
Leptotrichia sp. oral taxon 392	0	0	0	0	0	0
Leptotrichia sp. oral taxon 463	0.0023	0.0057	0.001	0.0025	0	0
Leptotrichia sp. oral taxon 498	0	0	0.0148	0.027	0	0
Leptotrichia trevisanii	0	0	0	0	0	0
Leptotrichia wadei	0	0	0.0197	0.0405	0	0
Mageeibacillus indolicus	0	0	0	0	0	0
Megasphaera genomosp. C1	0	0	0	0	0	0
Megasphaera micronuciformis	0.0101	0.0248	0	0	0	0
Methylobacterium haplocladii	0	0	0	0	0	0
Microbacterium sp. NDK-63	0	0	0	0	0	0

Microcella alkaliphila	0	0	0	0	0.0055	0.0135
Mogibacterium timidum	0	0	0	0	0	0
Mongoliitalea sp.	0	0	0	0	0.0069	0.017
Moryella sp. KHD1	0	0	0	0	0	0
Mycoplasma faucium	0.0031	0.0075	0	0	0	0
Mycoplasma hyorhinis	0	0	0	0	0.0064	0.0157
Mycoplasma salivarium	0	0	0	0	0	0
Natronohydrobacter thiooxidans	0	0	0	0	0	0
Neisseria bacilliformis	0	0	0	0	0	0
Neisseria elongata	0	0	0	0	0	0
Neisseria flava	0.0098	0.0241	0	0	0.0023	0.0056
Neisseria flavescens	0	0	0	0	0	0
Neisseria sp. 'ARUP UnID 432'	0	0	0	0	0	0
Neisseria sp. oral taxon 014	0	0	0	0	0	0
Neisseria sp. oral taxon 499	0	0	0.005	0.0123	0	0
Neisseria subflava	0	0	0	0	0	0
Nesterenkonia sp.	0	0	0	0	0	0
Nibricoccus aquaticus	0	0	0	0	0.023	0.0563
Nitrincola sp. E-044	0	0	0	0	0	0
Okadaella gastrococcus	0	0	0	0	0	0
Olsenella sp. F0004	0	0	0	0	0.0018	0.0044
Oribacterium sp. oral taxon 078	0	0	0	0	0	0
Paludibacter propionicigenes	0.0121	0.022	0	0	7e-04	0.0017
Parabacteroides sp. Marseille-P3160	0	0	0	0	0.0115	0.0281
Paracoccus sp.	0	0	0	0	0	0
Pararhodobacter sp.	0	0	0	0	0.0029	0.007
Parvimonas micra	0	0	0.0167	0.041	0.0028	0.0045
Parvimonas sp. canine oral taxon 102	0	0	0	0	0	0
Peptoanaerobacter [Eubacterium] yurii	0	0	0	0	0	0
Peptococcus sp. oral taxon 168	0	0	0	0	0	0
Peptoniphilus sp. oral taxon 836	0	0	0	0	0	0
Peptostreptococcus sp. CCUG 42997	0	0	0.0894	0.2189	0	0
Peptostreptococcus sp. oral taxon 113	0	0	0	0	0	0
Peptostreptococcus stomatis	0	0	0.0498	0.1221	0.0219	0.0536
Phocaeicola abscessus	0	0	0	0	0.0031	0.0076
Porphyromonas catoniae	0	0	0	0	0	0
Porphyromonas endodontalis	0	0	0.0168	0.0412	0	0
Porphyromonas gingivalis	0.0682	0.167	0.0017	0.0043	0.0125	0.0283
Porphyromonas pasteri	0	0	0	0	0	0
Porphyromonas sp. oral taxon 275	0	0	0	0	0	0
Porphyromonas sp. oral taxon 278	0	0	0	0	0	0
Prevotella baroniae	0	0	0	0	0	0
Prevotella conceptionensis	0	0	0.009	0.022	0	0
Prevotella dentalis	0.014	0.0343	0	0	0.0055	0.0135
Prevotella denticola	0.0311	0.0761	0	0	0.0077	0.0189
Prevotella genomosp. C1	0	0	0	0	0	0
Prevotella genomosp. P6	0	0	0.0057	0.014	0	0
Prevotella intermedia	0	0	0	0	0.0238	0.0582
Prevotella koreensis	0	0	0	0	0	0
Prevotella loescheii	0	0	0	0	0	0

Prevotella maculosa	0	0	0	0	0	0
Prevotella melaninogenica	0.016	0.0392	0.0368	0.0572	0.0075	0.0184
Prevotella micans	0	0	0	0	0	0
Prevotella multiformis	0	0	0	0	0	0
Prevotella nanceiensis	0	0	0	0	0	0
Prevotella oralis	0	0	0.0442	0.1083	0	0
Prevotella oris	0.0026	0.0064	0	0	0.0295	0.0669
Prevotella oulorum	0	0	0.0032	0.0078	0	0
Prevotella pallens	0	0	0.0209	0.04	0.005	0.0123
Prevotella salivae	0.0039	0.0096	0.0333	0.0816	0	0
Prevotella sp. 8404125	0	0	0	0	0	0
Prevotella sp. CM38	0	0	0	0	0	0
Prevotella sp. oral taxon 292	0	0	0	0	9e-04	0.0022
Prevotella sp. oral taxon 300	0.0086	0.021	0	0	0.0034	0.0084
Prevotella sp. oral taxon 303	0.0094	0.023	0	0	0	0
Prevotella sp. oral taxon 314	0	0	0	0	0	0
Prevotella sp. oral taxon 317	0	0	0	0	0	0
Prevotella sp. oral taxon 472	0	0	0	0	0	0
Prevotella sp. oral taxon 475	0	0	0	0	0	0
Prevotella sp. oral taxon 515	0	0	0	0	0.0042	0.0103
Prevotella sp. oral taxon G60	0	0	0	0	0	0
Prosthecomicrobium sp. 'ARUP UnID 533'	0	0	0	0	0	0
Pseudoleptotrichia goodfellowii	0	0	0	0	0	0
Pseudomonas tarimensis	0	0	0	0	0.0079	0.0125
Pseudomonas xinjiangensis	0	0	0	0	0.0074	0.0181
Pseudopropionibacterium massiliense	0	0	0	0	0	0
Pseudopropionibacterium propionicum	0	0	0	0	0	0
Pseudoramibacter alactolyticus	0	0	0	0	0.0782	0.1212
Pseudoruminococcus massiliensis	0	0	0	0	0	0
Rhodobacter sp. CCP-1	0	0	0	0	0.0021	0.0051
Roseomonas sp.	0	0	0	0	0.0134	0.0151
Rothia dentocariosa	0.0046	0.0114	0	0	0	0
Rothia mucilaginosa	0	0	0	0	0	0
Schaalia cardiffensis	0	0	0	0	0	0
Schaalia georgiae	0	0	0	0	0	0
Schaalia meyeri	0	0	0	0	0	0
Schaalia odontolytica	0.0366	0.0589	0	0	0	0
Schwartzia sp. canine oral taxon 042	0	0	0	0	0	0
Selenomonas artemidis	0	0	0	0	0	0
Selenomonas dianae	0	0	0	0	0	0
Selenomonas infelix	0	0	0.0043	0.0105	9e-04	0.0023
Selenomonas noxia	0	0	0.0013	0.0033	0	0
Selenomonas sp. oral taxon 126	0	0	0	0	0	0
Selenomonas sp. oral taxon 134	0	0	0.0084	0.0136	0	0
Selenomonas sp. oral taxon 137	0	0	0	0	0	0
Selenomonas sp. oral taxon 138	0	0	0	0	6e-04	0.0014
Selenomonas sp. oral taxon 149	0	0	0	0	0	0
Selenomonas sp. oral taxon 920	0	0	0	0	6e-04	0.0015
Selenomonas sp. oral taxon F19	0	0	0	0	0	0

Selenomonas sp. oral taxon F20	0	0	0	0	0	0
Selenomonas sp. oral taxon F21	0	0	0	0	2e-04	6e-04
Selenomonas sp. oral taxon F72	0	0	0	0	0	0
Selenomonas sp. oral taxon F81	0	0	0	0	0	0
Selenomonas sp. oral taxon F82	9e-04	0.0023	0	0	1e-04	3e-04
Selenomonas sp. oral taxon G00	0	0	0	0	0	0
Selenomonas sp. oral taxon G67	0	0	0	0	0	0
Selenomonas sp. oral taxon H64	0	0	0	0	2e-04	5e-04
Selenomonas sp. oral taxon H65	0	0	0	0	0	0
Selenomonas sp. oral taxon H66	0	0	0	0	0	0
Selenomonas sputigena	0	0	0.0115	0.0281	0	0
Shuttleworthia satelles	0	0	0	0	0	0
Slackia exigua	0.01	0.0244	0.03	0.0503	0.03	0.0734
Solobacterium moorei	0	0	0.005	0.0124	9e-04	0.0021
Staphylococcus aureus	0	0	0	0	0	0
Streptococcus anginosus	0	0	0.0778	0.1905	0.0238	0.0582
Streptococcus australis	0.01	0.0245	0	0	0.0044	0.0107
Streptococcus constellatus	0	0	0	0	0.006	0.0147
Streptococcus cristatus	0	0	0	0	0	0
Streptococcus downei	0	0	0	0	0	0
Streptococcus gordonii	0.0081	0.0197	0	0	0.0123	0.0301
Streptococcus gwangjuense	0	0	0	0	0	0
Streptococcus infantis	0	0	0	0	0	0
Streptococcus intermedius	0	0	0	0	0	0
Streptococcus mitis	0	0	0.0062	0.0152	0.0093	0.0169
Streptococcus mutans	0.0451	0.1106	0	0	0.1611	0.3946
Streptococcus oralis	0	0	0.0019	0.0047	0	0
Streptococcus parasanguinis	0.0011	0.0028	0.0124	0.0305	0.0022	0.0054
Streptococcus periodonticum	0.0127	0.0312	0	0	0	0
Streptococcus peroris	0	0	0	0	0	0
Streptococcus pneumoniae	0.0039	0.0095	0	0	0	0
Streptococcus salivarius	0	0	0.0106	0.0259	0	0
Streptococcus sanguinis	0	0	0	0	0	0
Streptococcus sp.	0	0	0	0	0	0
Streptococcus sp. 'ARUP UnID 614'	0	0	0	0	0	0
Streptococcus sp. 'ARUP UnID 639'	0	0	0	0	0	0
Streptococcus sp. 10aMclG2	0.0043	0.0106	0	0	0.0034	0.0084
Streptococcus sp. 10aVMg2	0	0	0	0	0	0
Streptococcus sp. 13aVMg2	0.001	0.0025	0	0	0	0
Streptococcus sp. A12	0	0	0	0	0	0
Streptococcus sp. AS14	0	0	0	0	0	0
Streptococcus sp. B2	0	0	0	0	0	0
Streptococcus sp. ChDC B366	0	0	0	0	0	0
Streptococcus sp. ChDC B519	0	0	0	0	0	0
Streptococcus sp. DP34	0	0	0	0	0	0
Streptococcus sp. JCM 5701	0	0	0	0	0	0
Streptococcus sp. JCM 5702	0	0	0	0	0	0
Streptococcus sp. NJ9704	0	0	0	0	0	0
Streptococcus sp. oral strain T4-E3	0	0	0	0	0.0058	0.0143
Streptococcus sp. oral taxon 064	0	0	0	0	0.0148	0.0363

Streptococcus sp. oral taxon 070	0	0	0	0	0	0
Streptococcus sp. oral taxon 071	0	0	0	0	0	0
Streptococcus sp. oral taxon 074	0	0	0	0	0.0018	0.0045
Streptococcus sp. oral taxon 431	0	0	0	0	0	0
Streptococcus sp. oral taxon C65	0	0	0.055	0.1347	0.0026	0.0063
Streptococcus sp. oral taxon E12	0	0	0	0	0	0
Streptococcus sp. Q28-2a	0	0	0	0	0	0
Streptococcus sp. THG-M4	0	0	0	0	0	0
Streptococcus sp. VA10345_05	0	0	0	0	0	0
Streptococcus vestibularis	0	0	0.0085	0.0208	0.0069	0.017
Tannerella forsythia	0	0	0	0	0	0
Tannerella sp. oral taxon 808	0	0	0	0	0	0
Tannerella sp. oral taxon HOT-286	0	0	0	0	0	0
Tessaracoccus aquimaris	0	0	0	0	0	0
Thermotalea metallivorans	0	0	0	0	0	0
Treponema denticola	0.0114	0.028	0.0072	0.0176	0	0
Treponema maltophilum	0.0058	0.0142	0	0	0	0
Treponema socranskii	0	0	0	0	0	0
Treponema sp. I	0	0	0	0	0	0
Treponema sp. IV	0	0	0	0	0	0
Treponema sp. oral taxon 237	0	0	0	0	0	0
Veillonella atypica	0	0	0	0	0	0
Veillonella dispar	0.0159	0.0251	0.0078	0.0158	0	0
Veillonella parvula	0.1667	0.4082	0	0	0	0
Veillonella ratti	0	0	0	0	0	0
Veillonella sp.	0	0	0	0	0	0
Veillonella sp. ICM51a	0.0019	0.0046	0	0	0.001	0.0019
Veillonella sp. oral taxon 158	0.0046	0.0113	0	0	0	0
Wandonia haliotis	0	0	0	0	0.0067	0.0164
